# Membrane-associated collagens with interrupted triple-helices (MACITs): evolution from a bilaterian common ancestor and functional conservation *in C. elegans*

**DOI:** 10.1186/s12862-015-0554-3

**Published:** 2015-12-14

**Authors:** Hongmin Tu, Pirkko Huhtala, Hang-Mao Lee, Josephine C. Adams, Taina Pihlajaniemi

**Affiliations:** Centre of Excellence in Cell-Extracellular Matrix Research, Faculty of Biochemistry and Molecular Medicine, Biocenter Oulu, University of Oulu, Aapistie 5, Oulu, FIN 90014 Finland; School of Biochemistry, University of Bristol, Biomedical Sciences Building, University Walk, Bristol, BS8 1TD UK

**Keywords:** Collagen, MACIT, Molecular phylogeny, Genome paralogy, Neuromuscular junction

## Abstract

**Background:**

Collagens provide structural support and guidance cues within the extracellular matrix of metazoans. Mammalian collagens XIII, XXIII and XXV form a unique subgroup of type II transmembrane proteins, each comprising a short N-terminal cytosolic domain, a transmembrane domain and a largely collagenous ectodomain. We name these collagens as MACITs (Membrane-Associated Collagens with Interrupted Triple-helices), and here investigate their evolution and conserved properties. To date, these collagens have been studied only in mammals. Knowledge of the representation of MACITs in other extant metazoans is lacking. This question is of interest for understanding structural/functional relationships in the MACIT family and also for insight into the evolution of MACITs in relation to the secreted, fibrillar collagens that are present throughout the metazoa.

**Results:**

MACITs are restricted to bilaterians and are represented in the Ecdysozoa, Hemichordata, Urochordata and Vertebrata (Gnathostomata). They were not identified in available early-diverging metazoans, Lophotrochozoa, Echinodermata, Cephalochordata or Vertebrata (Cyclostomata). Whereas invertebrates encode a single MACIT, collagens XIII/XXIII/XXV of jawed vertebrates are paralogues that originated from the two rounds of *en-bloc* genome duplication occurring early in vertebrate evolution. MACITs have conserved domain architecture in which a juxta-membrane furin-cleavage site and the C-terminal 34 residues are especially highly conserved, whereas the cytoplasmic domains are weakly conserved. To study protein expression and function in a metazoan with a single MACIT gene, we focused on *Caenorhabditis elegans* and its *col-99* gene. A *col-99* cDNA was cloned and expressed as protein in mammalian CHO cells, two antibodies against COL-99 protein were generated, and a *col-99*-bearing fosmid gene construct *col-99::egfp::flag* was used to generate transgenic *C. elegans* lines. The encoded COL-99 polypeptide is 85 kDa in size and forms a trimeric protein. COL-99 is plasma membrane-associated and undergoes furin-dependent ectodomain cleavage and shedding. COL-99 is detected in mouth, pharynx, body wall and the tail, mostly in motor neurons and muscle systems and is enriched at neuromuscular junctions.

**Conclusions:**

Through identification of MACITs in multiple metazoan phyla we developed a model for the evolution of MACITs. The experimental data demonstrate conservation of MACIT molecular and cellular properties and tissue localisations in the invertebrate, *C. elegans*.

**Electronic supplementary material:**

The online version of this article (doi:10.1186/s12862-015-0554-3) contains supplementary material, which is available to authorized users.

## Background

The extracellular matrix (ECM) of metazoans is an intricate, proteinaceous meshwork that underlies all epithelia and endothelia, and surrounds all connective tissue cells. It promotes cell adhesion, migration, differentiation and proliferation, and provides a supporting structure to which cells adhere. The complete genome sequences of multiple metazoans and especially domain arrangement studies have revealed the conservation and diversity of ECM proteins in metazoans [1, 2].

**Fig. 1:**
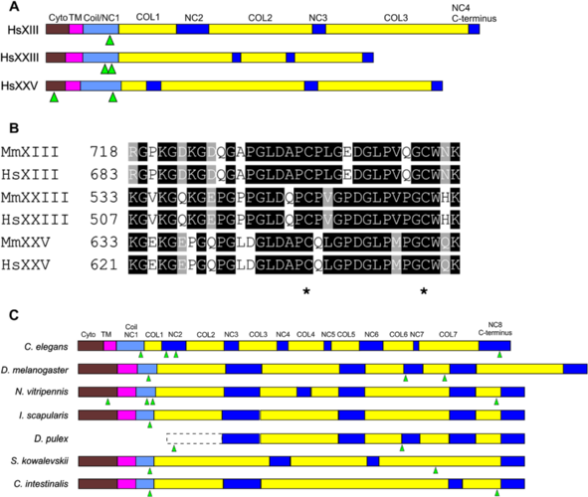
Domain architecture of MACIT collagens and conservation of the C-terminus. **a** Schematic diagrams of the domain organization of mammalian collagens XIII, XXIII and XXV, drawn to scale for the polypeptide lengths. **b** Multiple sequence alignment (MSA) of the C-termini of human and mouse MACITs. Black shading indicates residues conserved in more than 50 % of the sequences, grey shading indicates semi-conserved residues, white indicates non-conserved residues. Numbers refer to the starting amino acid position. Conserved cysteine residues are indicated with asterisks. **c** Schematic diagrams of the domain organization of MACIT collagens from invertebrates. In (**a**) and (**c**), collagenous (COL) domains are shown in yellow, non-collagenous (NC) regions in dark blue, the NC1 domain composed of coiled-coil sequences (Coil/NC1) in pale blue, the transmembrane domain (TM) in magenta, and the cytosolic domain (Cyto) in brown. Green arrowheads indicate putative furin cleavage sites

Collagens are one of the major classes of ECM macromolecules with multiple functions in the constitution and maintenance of the ECM of most animals [3]. Among the 28 collagen types known in mammals, collagens XIII, XXIII and XXV form a subgroup of structurally related collagens that have, so far, been studied only in mammals. These collagens, named here as MACITs (Membrane-Associated Collagens with Interrupted Triple-helices), are type II transmembrane proteins, composed of a short N-terminal cytosolic domain, a transmembrane domain and three collagenous domains (COL1-COL3), flanked and interrupted by non-collagenous sequences (NC1-NC4) (Fig. 1a) [4–6]. The ectodomains of the three collagens can be shed by furin convertases [5, 7, 8]. While the physiological functions and molecular mechanisms of MACIT collagens are not fully known, studies with genetically-engineered mouse models have suggested requirements for collagen XIII in the maturation of neuromuscular junctions (NMJ) [9, 10] and for collagen XXV in the development of the NMJ and the survival of motor neurons [11]. Moreover, collagen XXIII is up-regulated in human prostate and head and neck cancer progression [12–14] and collagen XXV is involved in amyloid β fibril aggregation and the formation of protease-resistant bundles, and therefore may regulate the development and progression of Alzheimer’s disease [5].

To complement the still scanty knowledge of the properties and functions of the MACITs in vertebrates we embarked on an investigation of the evolution of these proteins and their possible roles in invertebrates, with an experimental focus on *Caenorhabditis elegans. C. elegans* has over 150 collagen genes and most of these encode cuticle components, which are structurally similar to the FACIT (Fibril-Associated Collagens with Interrupted Triple-helices) collagens of vertebrates [15, 16]. There are also other collagen types known in *C. elegans*, especially those belonging to the metazoan basement membrane toolkit [2]. The collagen IV α-chain homologs EMB-9 and LET-2 are detected in the body wall muscle and some somatic cells of the gonad [17]. Mutations of *emb-9* or *let-2* cause lethality at the two-fold stage of embryogenesis [18]. The collagen XVIII homolog CLE-1 is expressed in body wall muscle and several neuronal subgroups [19]. *C. elegans* is also a useful model organism for functional analyses, especially of the nervous system, on account of the availability of genetic mutants [20–23] and novel, large scale genomic and proteomic tools [24, 25]. Recombineering-based transgene construction (*C. elegans* TransgeneOme) using a well-mapped fosmid (large genomic DNA, gDNA) clone library together with loss-of-function mutation rescue technology has provided a broad platform for the in vivo analysis of protein function in this animal [24, 26]. We report here for the first time that MACITs are widespread but not ubiquituous in bilaterians. We present molecular and phylogenomic analyses of the evolution of the MACIT collagens and demonstrate conservation of molecular, functional and tissue localization properties of *C. elegans* MACIT.

## Results

### Identification of MACIT homologues in many bilaterians

The domain architectures of mammalian collagens XIII, XXIII and XXV are shown in Fig. 1a. Typically, the transmembrane domain is followed extracellularly by a short coiled-coil region which assists in trimerisation [27, 28]. From multiple sequence alignment of human and mouse MACITs, we also noticed that the C-terminal 63 amino acids, especially the last 34 residues, are unusually highly conserved, both in species orthologues, and also between collagens XIII, XXIII and XXV. This sequence conservation includes two characteristically-spaced, completely conserved cysteine residues (asterisks, Fig. 1b). The functional role of this region is unknown. With regard to our goal of searching for MACIT proteins in other metazoans, our criteria for the identification of proteins related to collagens XIII, XXIII and XXV included: a) a predicted type II transmembrane topology; b) the presence of interrupted collagen triple helical regions in the predicted protein ectodomain, and c) sequence conservation of the C-terminal region motif including the cysteine residues.

**Fig. 2:**
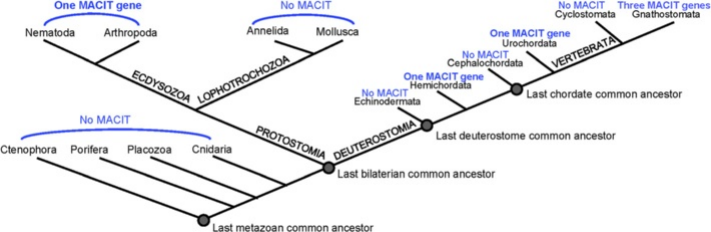
Schematic of MACIT evolution within the major lineages of the metazoan tree of life. The schematic evolutionary tree of metazoans demonstrates the major lineages of metazoans and the presence or absence of MACIT-encoding sequences identified in this study. The Gnathostomata (jawed vertebrates) are the only lineage in which more than one MACIT gene/genome was identified. Details of representative species in which MACITs were identified are given in Table 1

Sequences of human collagens XIII, XXIII and XXV were used first in systematic BLASTP and TBLASTX searches of the available NCBI genomic, cDNA or transcriptomic resources for birds, reptiles, amphibia, bony and cartilaginous fish, lampreys, urochordates, echinoderms, hemichordates and cephalochordates, protostome phyla, early-diverging metazoans and non-metazoans. Analyses were then expanded and “hits” validated as described in the Methods. This survey greatly expanded the dataset of recognized MACIT sequences and identified MACITs in many phyla in which MACITs were previously unknown. Accession numbers for MACITs from species representative of all the phyla in which MACITs were identified are given in Table 1. MACIT sequences were identified in multiple lineages of bilaterians, but not in any of the early-diverging metazoan phyla (Ctenophora, Porifera, Placazoa, Cnidaria), or several major protostome phyla (Annelida, Mollusca). Within deuterostomes, MACITs were not identified in the available species of Echinodermata (*Stronglyocentrus purpuratus),* Cephalochordata (*Branchiostoma floridae* or *Branchiostoma belcheri)*, or Cyclostomata (*Petromyzon marinus* and *Lethenteron japonicum*) (Fig. 2). MACITs were not identified in non-metazoan species.

**Table 1:** The dataset of MACIT protein sequences from protostomes and deuterostomes analysed in this study. Key: + sequence corrected versus expressed sequence tag

Phylum/Species/protein name	GenBank accession	Code name in MSAs and tree
Nematoda		
*Caenorhabditis elegans* MACIT/Col-99	NP_499869	Ce
*C briggsae* MACIT	A8WR59	Cb
Arthropoda		
*Drosophila melanogaster* MACIT	NP_001138061	Dm
*Nasonia vitripennis* MACIT	XP_008213977	Nv
*Tribolium castaneum* MACIT	EEZ97493.1	Tc
*Ixodes scapularis* MACIT	XP_002400454	Is
*Bombus impatiens* MACIT	XP_003486976	Bi
*Daphnia pulex* MACIT (incomplete N-end)	EFX66027.1	Dp
Hemichordata		
*Saccoglossus kowalevskii* MACIT	XP_006816441	Sk
Urochordata		
*Ciona intestinalis* MACIT	XP_002130216+	Ci
Vertebrata: Fish		
*Callorhinchus milii* XIII	XP_007896149	CmXIII
*Callorhinchus milii* XXIII	XP_007899591	CmXXIII
*Callorhinchus milii* XXV	XP_007887363	CmXXV
*Salmo salar* collagen XIII	NP_001167092	SsXIII
*Danio rerio* collagen XIII (partial)	XP_009305408	DrXIII
*Danio rerio* collagen XXIII	XP_009305980	DrXXIII
*Danio rerio* collagen XXV	XP_009301819	DrXXV
*Maylandia zebra* collagen XIII	XP_004572553	MzXIII
*Takifugu rubripes* collagen XXIII	XP_011609042	TrXXIII
*Takifugu rubripes* collagen XXV	XP_003967025	TrXXV
*Oryzias latipes* collagen XIII	XP_011482360	OlXXIII
*Oreochromis niloticus* collagen XXV	XP_005468213	OnXXV
*Latimeria chalumnae* collagen XXIII	XP_005999804	LcXXIII
*Latimeria chalumnae* collagen XXV	XP_005999645	LcXXV
Vertebrata: Amphibians		
*Xenopus tropicalis* collagen XIII	XP_002935816+	XtXIII
*Xenopus tropicalis* collagen XXV	NP_001123747	XtXXV
Vertebrata: Reptiles		
*Anolis carolinensis* collagen XXIII	XP_008103145	AcXXIII
*Anolis carolinensis* collagen XXV	XP_008110328	AcXXV
*Chrysemys picta bellii* collagen XIII	XP_008170226	CpbXIII
*Chrysemys picta bellii* collagen XXIII	XP_008164659	CpbXXIII
*Chrysemys picta bellii* collagen XXV	XP_008168359	CpbXXV
Vertebrata: Birds		
*Melopsittacus undulatus* collagen XIII	XP_ 005153749	MuXIII
*Taeniopygia guttata* collagen XIII	XP_004176296	TgXIII
*Gallus gallus* collagen XIII	XP-004942057	GgXIII
*Gallus gallus* collagen XXIII	XP_003642131	GgXXIII
*Gallus gallus* collagen XXV	XP_427455	GgXXV
*Pseudopodoces humilis* collagen XXV	XP_005518035	PhXXV
Vertebrata: Mammals		
*Equus caballus* collagen XIII	XP_003363528	EcXIII
*Equus caballus* collagen XXV	XP_005607984	EcXXV
*Canis lupus familiaris* collagen XXIII	XP_005626369	ClfXXIII
*Mus musculus* collagen XIII	NP_031757	MmXIII
*Mus musculus* collagen XXIII	NP_700442	MmXXIII
*Mus musculus* collagen XXV	NP_084114	MmXXV
*Ovis aries* collagen XIII	XP_004021682	OaXIII
*Ovis aries* collagen XXIII (partial)	XP_004009451	OaXXIII
*Ovis aries* collagen XXV	XP_004009675	OaXXV
*Homo sapiens* collagen XIII	NP_001123575	HsXIII
*Homo sapiens* collagen XXIII	NP_775736	HsXXIII
*Homo sapiens* collagen XXV	NP_942014	HsXXV

Within protostomes, single MACIT-encoding genes were identified in *C. elegans* and multiple other nematodes and in species from all classes of arthropods (Table 1, Fig. 2). In *C. elegans* the predicted MACIT protein (Fig. 1c), ORF F29C4.8, has been named COL-99 (Putative cuticle collagen 99) (www.wormbase.org). Expression of the gene has been confirmed at the mRNA level, but not proven by protein data [29]. Five alternative splicing variants have been predicted, although as yet without experimental confirmation (www.uniprot.org). We investigate COL-99 more deeply in the experimental sections of the Results.

In *D. melanogaster,* one predicted MACIT homologue was identified (Table 1 and Fig. 1c). Collagen α chain CG43342 is composed of 822 amino acids including one transmembrane domain close to the N-terminus and four interruptions within the collagenous ectodomain (Fig. 1c). The existence of a protein has not yet been proven but there is evidence at the level of transcription (www.uniprot.org). Four alternatively spliced isoforms have been predicted but these are yet to be confirmed experimentally [30]. In the modENCODE Temporal Expression Profile, peak expression of CG43342 has been detected at the 6-12 h embryonic stage through microarray studies [30]. Single MACIT-encoding genes were also identified in other Hexapoda (Table 1). The predicted MACITs of *T. castaneum*, *B. impatiens* and *N. vitripennis* MACITs are shorter proteins than *D. melanogaster* MACIT (in the range 638–682 residues) but are characterized by the same domain architecture including four interruptions of the collagenous regions (Table 1 and Fig. 1c). MACITs were also identified in other classes of arthropods, as exemplified by the chelicerate, *Ixodes scapularis*, and the crustacean, *D. pulex,* both of which contain four collagenous regions (Table 1 and Fig. 1c). The predicted *D. pulex* MACIT lacks cytoplasmic and transmembrane domains and thus the prediction appears to be incomplete at the N-terminal end (Fig. 1c).

Within deuterostomes, single MACIT encoding genes were identified in the hemichordate *Saccoglossus kowalevskii* and the urochordate *Ciona intestinalis* (Table 1, Fig. 2). The predicted polypeptides are of similar lengths to the mammalian MACITs. *S. kowalevskii* MACIT includes three collagenous regions and its coiled-coil region is only weakly predicted (residues 60-96, MARCOIL score of 0.2). *C. intestinalis* MACIT more closely resembles the domain architecture of mammalian collagens XIII, XXIII and XXV and contains three collagenous regions and a well-defined coiled-coil region (residues 73-104, MARCOIL score of 1.0) (Fig. 1c).

With regard to the encoding of MACITs in Gnathostomata (jawed vertebrates), no homologues were identified in the two available species of agnathans (jawless vertebrates), the lampreys *Petromyzon marinus* and *Lethenteron japonicum* [31, 32] (Fig. 2). This implies that the divergence of this lineage likely involved loss of the MACIT gene. Three predicted proteins corresponding to orthologues of collagens XIII, XXIII and XXV were identified in a cartilaginous fish, the elephant shark *Callorhinchus milii* [33]*,* and within bony (ray-finned) fish. In contrast, no collagen XIII orthologue was identified in the coelacanth *Latimeria chalumnae*, a species representative of the lobe-finned fish that are the closest group to the tetrapods [34], although collagen XXIII and XXV orthologues were present. No orthologues of collagen XXIII were identified in the available amphibian species, whereas orthologues to each of collagens XIII, XXIII and XXV were identified in species of reptiles, birds or mammals (Table 1).

### High conservation of the furin-cleavage sites and C-terminal region of MACITs contrasts with low conservation of the cytoplasmic domain

Release of the ectodomain by furin proteases is a well-established property of mammalian collagens XIII, XXIII and XXV [5–7]. Furin is widely expressed in eukaryotic cells. In *C. elegans* Furin-like proteinase KPC-1 has important roles in developmental functions [35, 36]. The furin cleavage site prediction software ProP is based on experimental results from the literature and computer data analyses [37]. In our study multiple sequence alignment of the newly identified MACIT sequences in conjunction with ProP prediction of furin cleavage sites for individual sequences identified that furin cleavage sites are also widely present in the MACITs of invertebrates (arrowheads in Fig. 1c). A predicted cleavage site within the NC1 region adjacent to the transmembrane domain was well-conserved. Variant sequences were identified in the invertebrate MACITs (see below). Unlike the mammalian MACITs, additional cleavage sites were predicted within the ectodomains of many of the invertebrate MACITS (Fig. 1c).

**Fig. 3:**
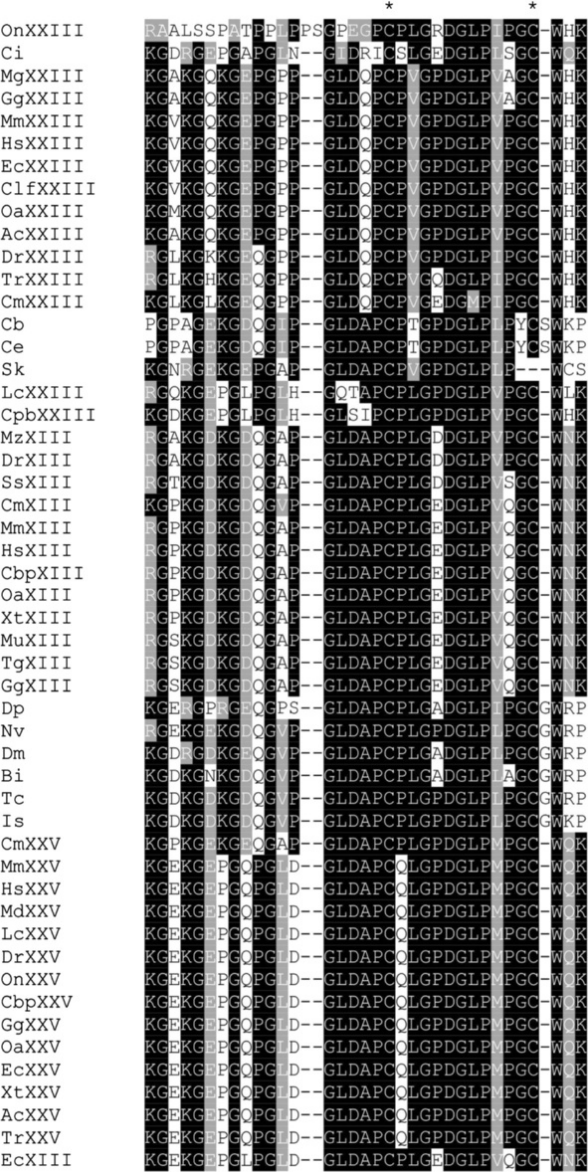
Multiple sequence alignment of the C-terminal sequences of representative MACITs. Shading code is as in Fig. 1b. Accession numbers and species code names are as in Table 1

**Fig. 4:**
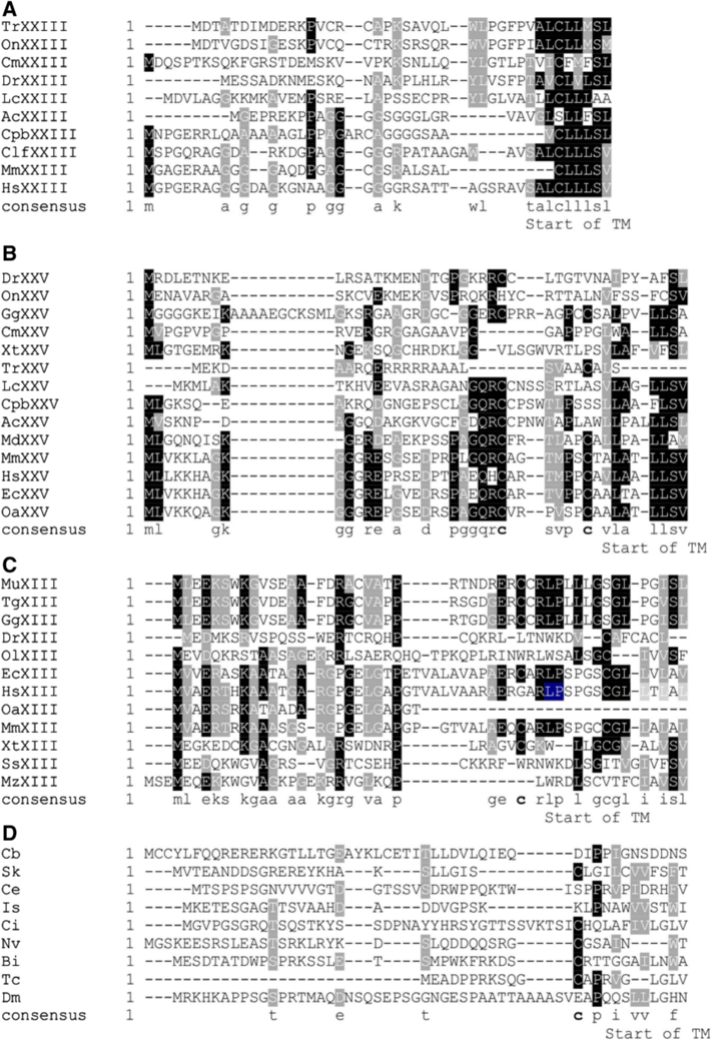
Multiple sequence alignments of the cytoplasmic domains of representative MACITS. Separate alignments were prepared for collagens XXIII (**a**), XXV (**b**), XIII (**c**), and invertebrate MACITs (**d**). A consensus sequence (the aa represented in at least 50 % of the sequences) is shown below each set with the conserved cysteines bolded. Shading code is as in Fig. 1b. Species code names are as in Table 1

The C-terminal region of MACITs includes triple helical motifs and a final non-collagenous region that are extremely highly conserved across the representative MACITs (Fig. 3). With the exception of *C. intestinalis*, MACITs from invertebrates typically differ in the C-terminal three amino acids, WKP, WCS, or WRP instead of W(H/N/Q)K, and the C-terminal domain extends about 30 residues beyond this in the MACITs of invertebrates (shown for *C. elegans* in Additional file 1). In contrast, the N-terminal regions, corresponding to the cytoplasmic domain, are weakly conserved and aligned poorly when all the MACITs in our dataset were entered in a multiple sequence alignment (unpublished observation). However, when collagen XIII, XXIII and XXV sequences were examined separately as sets of species orthologues, elements of conservation were apparent, although the consensus for collagen XXIII was much weaker than those derived for collagen XIII or XXV (Fig. 4a–c). The cytoplasmic domains of collagens XIII and XXV include one or two conserved cysteine residues close to the transmembrane domain (bold in consensus in Fig. 4b and c). Possibly, these residues are targets for palmitoylation [38]. The cytoplasmic domains of invertebrate MACITs are variable in length and show very little sequence conservation. A juxta-membrane cysteine is present in a number of these sequences, although not in nematode, *D. melanogaster,* or *I. scapularis* MACITs (Fig. 4d).

### The evolution of MACITs has involved expansion to a gene family in vertebrates

It is well-established that many gene families of vertebrates consist of paralogous genes that arose during the two rounds of large-scale, *en bloc* genome duplication that took place early after the divergence of vertebrates; these events are considered to have taken place before the divergence of cartilaginous fish [39, 40]. These events have resulted in the existence of large, paralogous chromosomal regions within the genomes of vertebrates [41] that can be used to derive information on the relationships between members of vertebrate gene families. Therefore, we next examined whether *COL13A1*, *COL23A1* and *COL25A1* are located within paralogous regions of the human genome. The human genome is suitable for this analysis because the rate of DNA rearrangement is relatively slow [42].

**Fig. 5:**
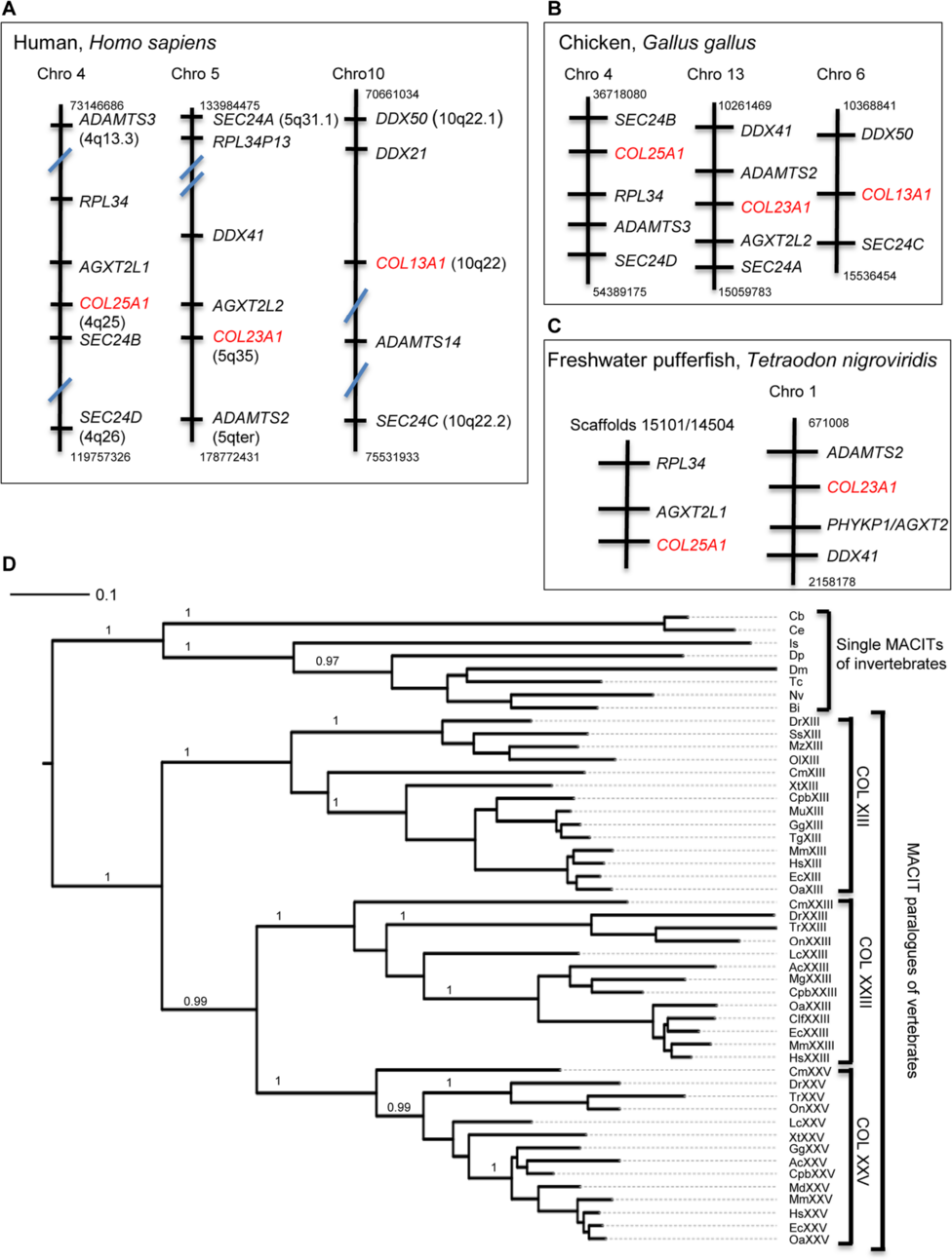
Phylogenomic analyses of the MACITs. *COL13A1*, *COL23A1* and *COL25A1* are located in paralogous regions of the human (**a**), chicken (**b**), and freshwater pufferfish (**c**), genomes. In **a**-**c**, each stick diagram represents the region of the chromosome in the vicinity of the relevant MACIT gene and each horizontal line represents a gene; for simplicity of presentation, only the conserved syntenic genes have been included. Numbers above and below each diagram refer to the start and end positions of the first and last genes presented, respectively, on the chromosome in bases. In (**c**), *T. nigroviridis COL25A1* is unmapped in the genome, but is located within the same two over-lapping scaffolds as two of the genes that are syntenic in human and chicken. **d**, Phylogenetic relationships of MACIT proteins. Bootstrap values above 0.95 are taken to indicate stability of a branchpoint and are shown for the major nodes. Scale bar indicates substitutions/site. Species code names in (**d**) are as in Table 1

Initial assessment through the database of paralogons in the human genome [43] identified that the chromosomal regions 4q25, 5q35 and 10q22 indeed share blocks of paralogy. Detailed analysis of shared paralogous genes between these regions based on the human reference genome (NCBI assembly GRCh38.p2), identified that, in addition to *COL13A1*, *COL23A1* and *COL25A1*, genes encoding members of the Sec24 gene family and three members of the ADAMTS gene family are located within each of these chromosomal regions (Fig. 5a). ADAMTS2, -3 and -14 are known members of a well-recognized ADAMTS sub-family on the basis of their protein sequence characteristics [44]. Support for the paralogy of these chromosomal regions is also provided by the presence of several paralogous gene pairs: for example *AGXT2L1* and *AGXT2L2* on chromosomes 4 and 5, respectively, or members of the *DDX* gene family on chromosomes 5 and 10 (Fig. 5a).

To confirm that these relationships at the level of the genome did indeed represent the results of the *en bloc* genome duplication early in the vertebrate lineage, we also examined species representative of earlier diverging vertebrates: the chicken, *G. gallus,* and the freshwater pufferfish, *T. nigroviridis*, for possible conservation of neighboring genes around the *COL13A1*, *COL23A1* and *COL25A1* loci. Conservation of synteny was apparent for the regions around all three genes in the chicken (Fig. 5b). In the pufferfish, *COL13A1* is unmapped and genes orthologous to *ADAMTS14*, *DDX50*, etc., could not be identified on the same scaffold. Conservation of synteny on chromosome 1 was apparent for the gene neighbours of *COL23A1. COL25A1* is unmapped, however, *RLP34* and *AGXT2L1* are located on the same genomic scaffold, thus demonstrating conservation of synteny for this locus (Fig. 5c). The conservation of synteny across these three lineages confirms that these paralogous regions arose from the two rounds of large-scale genome duplication early in the vertebrate lineage.

Although these data clearly demonstrated that *COL13A1*, *COL23A1* and *COL25A1* are located in paralogous regions of vertebrate genomes, the depth of information on paralogous neighbouring genes was not sufficient to distinguish between possible models of the evolutionary relationships between *COL13A1*, *COL23A1* and *COL25A1* with reference to the two rounds of genome duplication. Therefore, molecular phylogenetic studies of protein sequence relationships were undertaken.

### Molecular phylogeny of MACIT proteins

To develop a clear model of the evolution of the MACIT gene family in vertebrates, we next analysed their molecular phylogeny on the basis of their protein sequences. In initial tree-building tests, *C. intestinalis* and *S. kowlevskii* MACITs consistently grouped with the arthropod MACITs, likely due to long-branch attraction, and destablised the trees. Therefore, these two sequences were not included in the final dataset for tree-building (unpublished observation). Further tests consistently demonstrated very similar topologies for trees based on multiple sequence alignments prepared in webPRANKS [45] or MUSCLE [46]. Only the webPRANK-based results, that consistently had better statistical support, are shown here. The rooted tree based on 49 MACIT sequences and rendered from phylogenetic inference analysis and tree-building with the maximum-likelihood method, PhyML, demonstrated that the MACITs of invertebrates form a clade separate from collagens XIII, XXIII or XXV, and also identified that collagen XXIII and XXV are more closely related to each other than to collagen XIII. The root of the tree was placed between the invertebrate MACITs and the MACITs of vertebrates (Fig. 5d).

### *C. elegans* MACIT mRNA undergoes extensive alternative splicing

Because *C. elegans* is a well-established invertebrate model for developmental and functional studies of the neuronal system [22] and with regard to the novel possibility to study protein expression and function in a metazoan with a single MACIT gene, this species was chosen for experimental studies. To confirm the predicted protein sequence data the protein coding region of the *col-99* gene was cloned using PCR on synthesized cDNA produced by reverse transcription of mRNA. The full-length nucleotide sequence was obtained by DNA sequencing and the predicted amino acid sequence was compared with other available COL-99 sequences in the *C. elegans* database (www.wormbase.org) (Additional file 1). It should be noticed that the five COL-99 isoforms in the database are only in part confirmed by high-throughput RNA-seq in the modENCODE project for integrative analysis of the *C. elegans* genome [29]. The complete cDNA sequences for these isoforms have not been determined experimentally. All the clones acquired from the cDNA cloning in the current study were of a new type, named as COL-99f (BankIt1764273 col-99 KM875546). We determined that COL-99a lacks exon 4, COL-99c lacks exons 4 and 8, COL-99d lacks exons 4 and 12, COL-99e lacks exons 4 and 16, and the newly identified COL-99f lacks exons 4, 12, 16. A schematic diagram for the six isoforms is presented in Additional file 2.

### Furin-mediated shedding is a conserved property of *C. elegans* COL-99

**Fig. 6:**
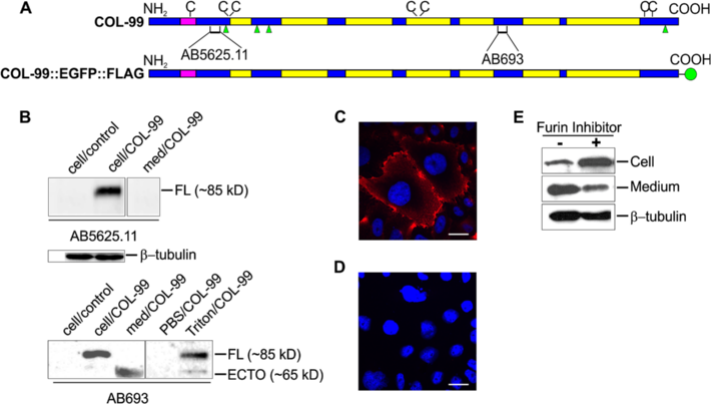
Characterization of the MACIT protein COL-99 of *C. elegans*. **a** Schematic structure of the COL-99 and COL-99::EGFP::FLAG polypeptides. The domain structure is presented as in Fig. 1 and cysteines (**c**) are also indicated. The regions used as antigens for the antibodies AB5625.11 and AB693 are marked. The C-terminal tag of COL-99::EGFP::FLAG is shown as a green circle. **b** Western blot analysis of subcellular localization of recombinant COL-99 in CHO cells. From the left to right: control lysate, total lysate of CHO cells expressing recombinant COL-99, concentrated medium sample, in the upper and lower panels detected with antibody AB5625.11 (*upper*) or AB693 (*lower*); and additional PBS-soluble proteins, and Triton X-100-extracted protein fraction from CHO cells expressing COL-99 in the lower panel only detected with AB693. **c** Immunofluorescent staining of CHO cells expressing COL-99. The signal was detected by antibody AB5625.11 and secondary anti-rabbit IgG conjugated with AlexaFluor 594. **d** Control staining with the secondary antibody only. Bars, 20 μm. **e** Inhibition of COL-99 ectodomain shedding by Furin Inhibitor I. Right lane, COL-99 expressing CHO cell cultures treated with 2 μM Furin Inhibitor I. Left lane, cells treated with an equivalent volume of methanol as solvent control. β-tubulin was used as a loading control in (**b**) and (**e**)

The *C. elegans* MACIT COL-99 variants are characterized by 7 interrupted collagenous domains (Additional file 1; Fig. 1c). Moreover, there are four putative furin-like protease cleavage sites with a consensus motif RXXR. The cleavage prediction scores calculated with the software ProP differ between RRVR^104^ (0.353), RRPR^137^ (0.132) RKMR^153^ (0.470), and RRKR^648^ (0.556). RRVR^104^ is located in the first, NC1, non-collagenous domain corresponding to a cleavage site that is widely conserved (Fig. 1c), but RKMR^153^ is predicted as a more likely furin cleavage site. The C-terminal putative cleavage site (RRKR^648^) is not present in mammalian MACITs (Additional file 1 and Fig. 1a, c). It is noticeable that in human collagen XIII the single furin cleavage site, RRRR^107^, has a prediction score of 0.649. This cleavage site has been confirmed in our previous study of recombinant collagen XIII expressed in insect cells [7]. In human collagen XXIII, there are two putative furin cleavage sites RLLR^97^ and RTAR^110^ in the NC1 domain, both with relatively low cleavage prediction scores of 0.285 and 0.315, respectively. In human collagen XXV, one site, REPR^16^, with a score of 0.511 is located in the cytosolic domain, whereas another site, RIAR^112^, is located within the NC1 domain, but with a lower score of 0.328. *C. elegans* MACIT contains 7 cysteines, of which the positions of two in the NC1 domain and two in the NC8 domain are conserved with mammalian MACITs (Fig. 6a). Cysteines in the transmembrane domain and the central non-collagenous portion have similar positions to mammalian MACITs (Additional file 1).

To study the biochemical characteristics of the COL-99 protein we expressed it as a recombinant protein in mammalian CHO cells. Using as a template RNA extracted from the worm line *col-99::egfp::flag* (for details on the worm line, see the section Expression of col-99 in C. elegans is highest at the L1-L2 larvae stages of development), we cloned the *col-99* cDNA (without the EGFP and FLAG tags as in the fosmid these were codon-optimized for *C. elegans* gene expression), into a mammalian cell expression vector, which then was used to transfect CHO cells. For detection of COL-99 protein, two new antibodies against COL-99 protein were generated: the first antibody, termed AB5625.11, being raised against a peptide in the NC1 domain before the predicted furin cleavage site, and the second antibody, AB693, against a peptide located in the predicted ectodomain region (Fig. 6a). In western blots of whole cell extracts under a reducing condition, the antibody AB5625.11 detected a band of 85 kDa in CHO cells expressing COL-99, whereas control CHO cells expressing EGFP gave no signal (Fig. 6b). This band can be expected to represent monomeric (single-chain) full-length COL-99, because a furin-based proteolytic cleavage fragment would correspond to a small N-terminal peptide (~17 kD) detected by AB5625.11. Moreover, no signal was seen in the medium with this antibody, demonstrating that the full-length molecules are retained in the cell fraction due to the transmembrane domain (Fig. 6b). Next, western blotting with antibody AB693 was performed, because this antibody should make it possible to identify both full-length and shed molecules. Indeed, the antibody AB693 identified a band of 85 kDa in the lysates of COL-99-expressing CHO cells, as also seen with AB5625.11. In addition, a band of ~65 kDa was detected in the corresponding medium (Fig. 6b), suggesting cleavage at the N-terminal furin sites. In a further experimental design, the proteins in the COL-99-expressing CHO cells were extracted sequentially by PBS, and then PBS containing 1 % Triton X-100. Only the latter treatment resulted in extraction of the COL-99 protein (Fig. 6b), the majority of which corresponded to the full-length protein and only minor amounts to the shed form, characteristic of a membrane-associated protein. This need for detergent-based extraction of the cell-associated collagen XIII protein was also reported for mammalian MACIT collagens [7]. The cell membrane location of COL-99 was also defined by immunofluorescence staining of CHO cells stably transfected with the plasmid encoding *col-99* (Fig. 6c & d).

According to the molecular mass difference of the full-length and the shed forms of COL-99 (Fig. 6b) and the PrOP 1.0 scores of predicted furin cleavage sites, COL-99 can potentially be shed by furin cleavage at RRVR^104^/RKMR^153^ and RRKR^648^, resulting in an ectodomain composed of 544 or 495 amino acids starting from N^105^ or A^154^, respectively, to R^648^ (Additional file 1 and Fig. 6b). Indeed, addition of Furin Inhibitor I to the culture medium of CHO cells expressing COL-99 resulted in a clear reduction in the level of COL-99 in the culture medium (~70 %), while the COL-99 signal in the cell fraction was reciprocally elevated (Fig. 6e). While the data demonstrate furin cleavage at one or more of the N-terminal sites, use of the less conserved C-terminal site, present in *C. elegans* and other non-vertebrate MACITs, remains to be proven.

### Expression of *col-99* in *C. elegans* is highest at the L1-L2 larvae stages of development

We next used the fosmid-based *C. elegans* line *col-99::egfp::flag* to study the expression and localization of COL-99 protein localization in the animals, on the basis that the fosmid-based expression is likely close to the native condition [24]. The col-99-bearing fosmid gene construct col-99::egfp::flag was used to generate transgenic *C. elegans* lines. From two separate gene transfers we obtained three lines with stable genomic integration. To test the construction strategy we also performed ballistic transformation of another fosmid encoding the *C. elegans* integrin gene, *pat-3* (ZK1058.2, WormBase ID is WBGene00003930), chosen as a positive control because data on PAT-3 protein localization in body muscle by antibody staining is available [47].

**Fig. 7:**
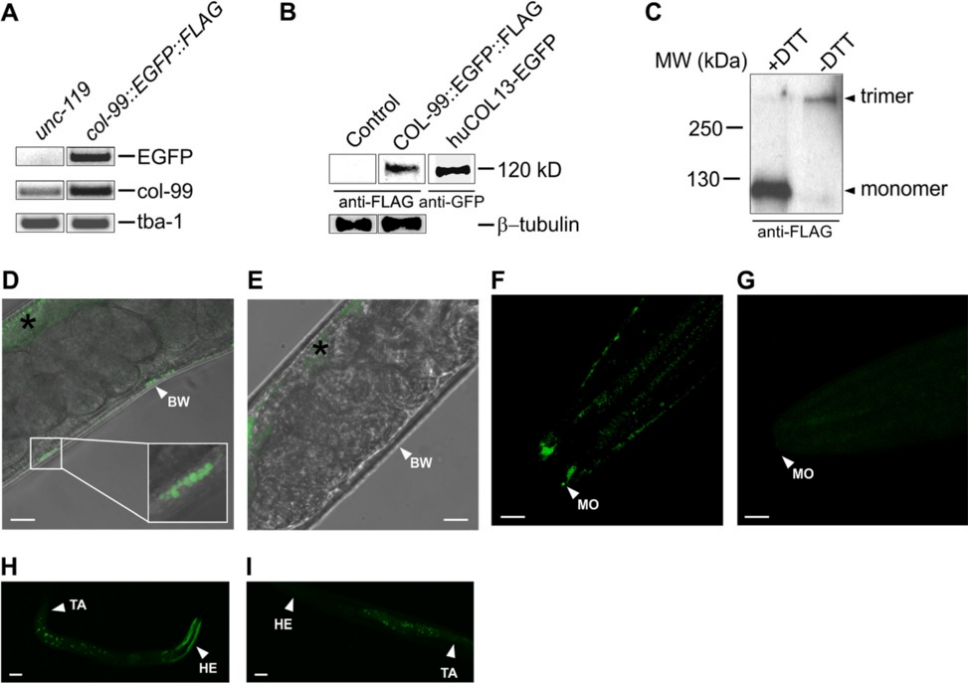
Expression of COL-99::EGFP::FLAG in *C. elegans*. **a** Verification of the transgenic worm line col-99::egfp::flag by RT-PCR. Primers are specific for cDNA of EGFP, col-99 and the reference gene tba-1 respectively. **b** Western blot analysis of the EGFP- and FLAG-tagged COL-99 protein in *C. elegans* lysates resolved under reducing conditions. Lanes from left to right are lysates from worm lines unc-119, col-99::egfp::flag, and from CHO cell expressing human collagen XIII with an EGFP-tag. β-tubulin was used as a loading control. **c** Western blot of col-99::egfp::flag worm lysate with or without DTT reducing agent treatment. Arrows indicate the COL-99::EGFP::FLAG protein in monomeric (~120 kDa) or trimeric (>300 kDa) forms. The detection antibody was anti-FLAG. **d** Localization of COL-99::EGFP::FLAG at the worm body wall (BW) imaged by confocal microscopy. **e** Green fluorescence imaging of the control line *unc-119*. The asterisks in (**d**) and (**e**) indicate autofluorescence produced by the bacteria in the *C. elegans* intestine. **f** In vivo imaging of an adult worm mouth (MO) part. The 2D image was converted from a 3D confocal scanning. **g** Image of the same region in a control *unc-119* worm. **h** Whole worm imaging of a L1 larva (HE = head, TA = tail). **i** Control image of an *unc-119* L1 larva. Bars in (**d**)-(**i**), 10 μm. About ten worms of each type were imaged in total

Fosmid-based gene transcription was confirmed by rescue of an *unc-119* loss-of-function mutation in the *C. elegans* strain HT1593 (called here the *unc-119* line and used here as a control) and by RT-PCR using primers specific for fosmid-based transcripts. All three *col-99::egfp::flag* lines showed the correct size of a DNA fragment from the EGFP cDNA by RT-PCR, and one of the strains was selected for further analysis (Fig. 7a). RNA expression analysis revealed endogenous *col-99* transcripts in the *unc-119* line and a more prominent band in the new *col-99::egfp::flag* line, suggestive of a higher expression level when the transgene augments the endogenous expression (Fig. 7a). In western blot analysis of nematode lysates, the fusion protein COL-99::EGFP::FLAG showed a molecular mass of ~120 kD, that is remarkably similar to that of the recombinant human collagen XIII-EGFP fusion protein expressed in CHO cells (Fig. 7b). Moreover, under non-reducing conditions, the protein COL-99::EGFP::FLAG was detected as a trimer (Fig. 7c). Antibodies to either the GFP- or the FLAG- tag were able to detect the fusion protein in *col-99::egfp::flag* worm lysates, but the anti-FLAG monoclonal antibody showed a more specific signal (Additional file 3). It should be noted that in the construction of *col-99::egfp::flag* the tag part including the EGFP, FLAG and the linker peptides accounts for a molecular mass of 35 kD.

The adult worms showed very weak *col-99* expression (Fig. 7d, f and h) in vivo compared to the *pat-3::egfp::flag* line (Additional file 4). The weak fluorescent signals were detected only in some locations at the body wall (Fig. 7d) and the mouth (Fig. 7f). These signals often occurred in clusters. The control line *unc-119* was negative in the direct EGFP fluorescence imaging (Fig. 7e and g), except for green auto-fluorescent signals in the intestine that are derived from the *E. coli* food and are seen in both the *col-99* transgenic and the *unc-119* line (asterisks in Fig. 7d and e). Interestingly, the head part did show prominent signals in younger worms, especially in L1-L2 larvae (Fig. 7h). This signal was specific to the *col-99::egfp::flag* line (Fig. 7i).

### Col-99 is expressed in the *C. elegans* neuronal and muscle systems

**Fig. 8:**
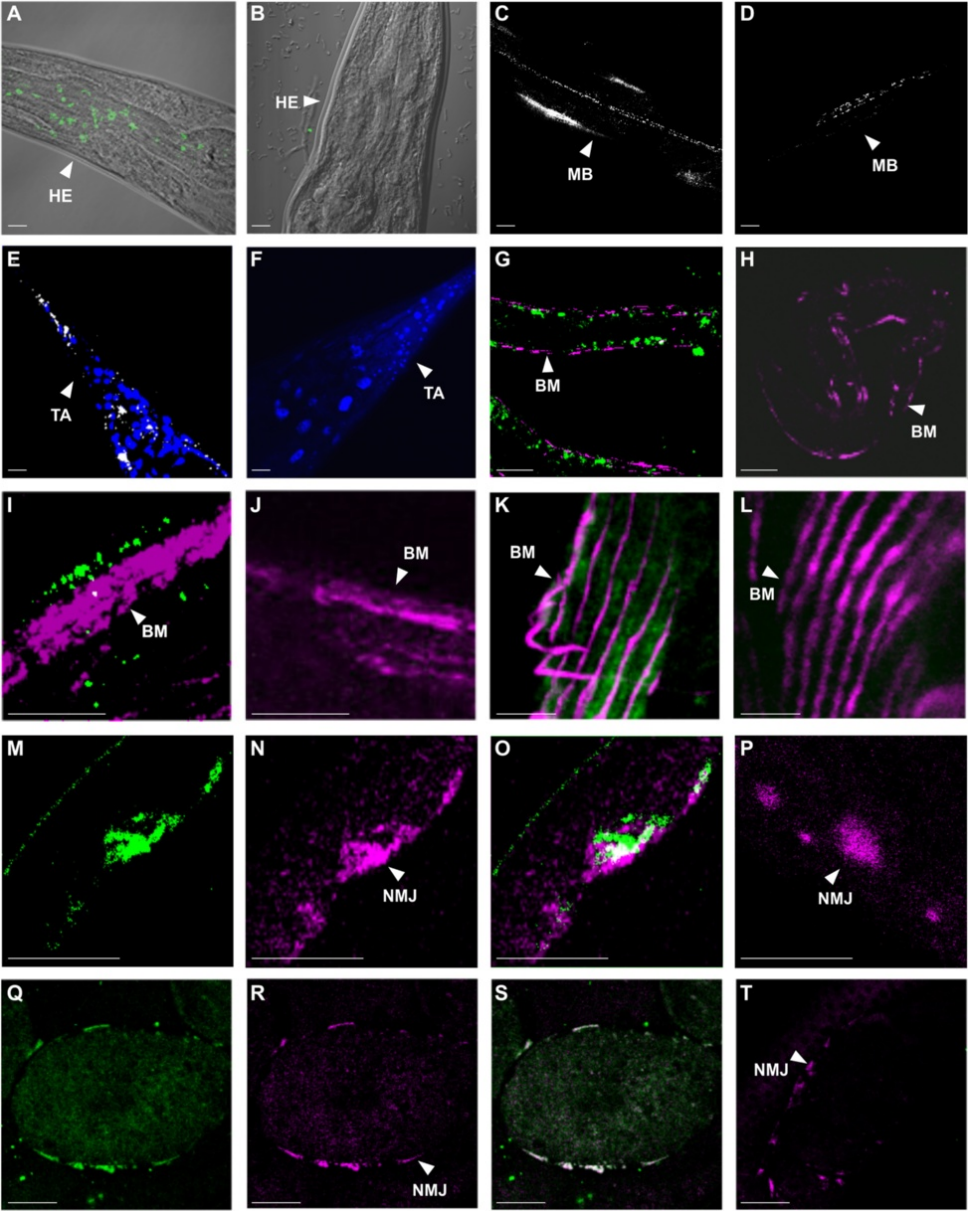
Localization of COL-99::EGFP::FLAG at NMJs and in other tissues. **a** Detection of COL-99::EGFP::FLAG expression in the head part of an adult *C. elegans*. Whole mount immunofluorescence staining was performed with a rabbit anti-GFP antibody and AlexaFluor 488-conjugated anti-rabbit IgG. **c** Detection of COL-99::EGFP::FLAG in the middle body part. The specimen was prepared by a freeze-crack treatment and a mild PFA fixation. The detection antibody was rabbit anti-GFP. **e** Staining of methanol/acetone fixed tail (TA) section of a *col-99::egfp::flag* worm with rabbit anti-GFP (*white*) and DAPI (*blue*). The green fluorescence detected with AlexaFluor 488-conjugated secondary antibody was converted to white to enhance the sensitivity. **g** Double staining of an L1 larva with rabbit anti-GFP (*green*) and mouse anti-myosin (*magenta*). The image was converted from a 3D Z-scan and signal was adjusted by Image J software. **i** Whole-mount immunofluorescent staining of a *col-99::egfp::flag* adult worm with rabbit anti-GFP (*green*) and mouse anti-myosin (*magenta*). **k** Immunofluorescent staining of a freeze-crack section of worm muscle after mild PFA fixation with rabbit anti-GFP (*green*) and mouse anti-myosin (*magenta*). **m**-**o** Immunofluorescent staining of a *col-99::egfp::flag* worm after freeze-crack treatment with rabbit anti-GFP (*green* in **m** and **o**) and α-bungarotoxin (*magenta* in **n** and **o**) showing NMJ localization. **o** Merged from (**m**) and (**n**). **q**-**s** Immunofluorescent staining of *col-99* embryos with rabbit anti-GFP (*green* in **q** and **s**) and α-bungarotoxin (*magenta* in **r** and **s**). **b**, **d**, **f**, **h**, **j**, **l**, **p**, and **t** are negative control staining of unc-119 worms for (**a**), (**c**), (**e**), (**g**), (**i**), (**k**), (**o**), and (**s**) respectively. Bars, 5 μm in **a**-**h**, **k**-**l** and **m**-**p**, 10 μm in **i**-**j**, and 20 μm in **q**-**t**. HE, head; MB, middle body; TA, tail; BM, body muscle; NMJ, neuromuscular junction

To obtain more detailed data on COL-99 localization, we performed whole mount immunofluorescence staining of the *col-99::egfp::flag* worms at different developmental stages, using both methanol/acetone and PFA fixation methods. With anti-GFP staining, COL-99::EGFP::FLAG was detected as spots or clusters in pharyngeal muscle or brain sites in methanol/acetone-fixed specimens (Fig. 8a) whereas the *unc-119* control line was negative (Fig. 8b). In PFA-fixed specimens, COL-99 was also detected in the middle body (Fig. 8c) and in the tail (Fig. 8e), whereas the control *unc-119* line was negative at these tissue sites (Fig. 8d and f).

We next compared the COL-99::EGFP::FLAG signals with different molecular markers for muscles or neurons. A spot-like appearance of COL-99::EGFP::FLAG was detected by immunofluorescent staining with anti-GFP and this was adjacent to the body muscle marker myosin in L1-L2 larvae (magenta in Fig. 8g), suggesting that COL-99::EGFP::FLAG associates with motor neurons. As expected, the control line was negative for GFP staining (Fig. 8h). In adult worms, spot-clustered staining of COL-99::EGFP::FLAG (green) was detected weakly on the body wall along the muscle fibers (magenta) (Fig. 8i) and was specific to the transgenic line (Fig. 8j shows the *unc-119* line). However, the overall number of COL-99::EGFP::FLAG-positive sites was lower than in the larvae. Weak expression of COL-99::EGFP::FLAG (green) in body muscle was detected in a sagittal freeze-cracked section after mild, short fixation with PFA, but this staining did not co-localize with the muscle marker myosin (magenta) (Fig. 8k). This COL-99 staining was specific because it was not detected in the control line *unc-119* (Fig. 8l). However, clear co-localization of COL-99::EGFP::FLAG (green) and the NMJ marker α-bungarotoxin (magenta) was observed in the body muscle of adult worms (Fig. 8m–o) and in late-stage embryos (Fig. 8q–s). No COL-99::EGFP::FLAG staining was detected in the respective *unc-119* negative controls (Fig. 8p and t).

## Discussion

Our investigations of MACIT proteins demonstrate for the first time the extent of the MACIT family in bilaterian animals. The collection of a large dataset of protein sequences has enabled an in-depth analysis of conserved features of this unique sub-family of transmembranous collagens. With new tools and antibodies for *C. elegans* MACIT, we have made novel observations on the conservation of functional properties between this MACIT from invertebrates and the mammalian MACIT family.

Overall, the phylogenetic distribution of MACITs in extant species leads to the inference that a MACIT-encoding gene originated in the last common bilaterian ancestor, but has been lost from multiple lineages within both protostomes and deuterostomes. Secreted forms of collagens, exemplified by the fibrillar collagens, are present throughout all metazoans [48], thus the simplest inference is that MACIT collagens evolved far later than secreted collagens. The data on conservation of paralogous locations of *COL13A1*, *COL23A1* and *COL25A1* in the human, chicken and freshwater pufferfish genomes support the model that the MACIT gene family of vertebrates debuted during the *en-bloc* genomic duplications early in the evolution of vertebrates. Because only three MACIT genes are present in modern vertebrates, the data imply that a fourth paralogon resulting from the second round of duplication was lost early in the divergence of vertebrates. Additional gene losses appear to have occurred subsequently in some vertebrate lineages. Whereas orthologues of collagen XXV were identified in all classes of vertebrates, collagen XIII appears to have been lost in the lobe-finned fish lineage and collagen XXIII to have been lost from the available amphibian species. Information on additional species of lobe-finned fish or amphibians would be needed to substantiate these interpretations.

**Fig. 9:**
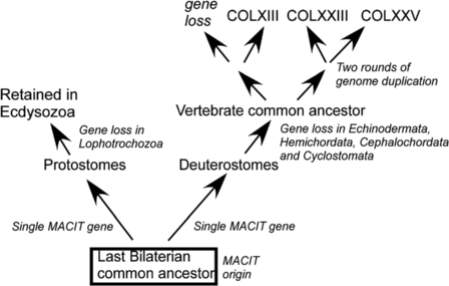
Model for the evolution of MACIT collagens. See text for details

Together, these data and the data on the phylogenetic distribution and molecular phylogeny of MACITs in extant metazoans, lead us to propose to the following model for MACIT evolution (Fig. 9). It is inferred that the MACIT ancestral gene originated in the last bilaterian common ancestor, and has been conserved as a single gene in Ecdysozoa (arthropods and nematodes), Hemichordata and Urochordata, whereas the gene has been lost in the Lophotrochozoa and certain deuterostome phyla (Echinoderma, Cephalochordata and Cyclostomata). Indeed, lampreys are known to have multiple distinct proteins within their ECM, including non-collagenous cartilaginous ECM proteins such as lamprin [49]. The two rounds of en-bloc genome duplication that took place early in the vertebrate lineage then led to the origin of a family of MACIT paralogues, with loss of a putative fourth family member soon after the initial gene duplication events. The observed closer sequence relationship of collagen XXIII and XXV proteins are explained most parsimoniously by the model that these represent the extant forms arising from one pair of duplicated genes produced in the second round of genome duplication (Fig. 9). Thus, collagen XXIII and XXV genes have had a shorter time for divergence from each other than from the collagen XIII gene. The formation of a distinct clade by collagen XIII in the phylogenetic tree is taken to indicate that collagen XIII represents the single remaining member from the second pair of duplicated genes (Fig. 9). An alternative or additional possibility is that collagen XIII may be under different selection pressures and evolving at a different rate from collagens XXIII and XXV.

Through the phylogenetic studies, we identified *col-99* as the sole *C. elegans* homologue of the mammalian MACIT collagens. To examine the possibility of functional conservation between an evolutionarily distant MACIT with the mammalian MACITs, we focused on *col-99*. Alternative splicing of collagen XIII with complicated exon deletions was discovered in our early gene transcription studies in human and mice. The untranscribed exons encode collagenous, or non-collagenous sequences, as well as partly collagenous and partly non-collagenous domains. All splice variants maintain the transmembrane domain, furin cleavage site and the C-terminal conserved peptide [50–52]. Due to the limited studies of collagen XXIII and XXV, alternative splicing of transcripts from these genes has not yet been adequately documented. Only two splicing variants of collagen XXV are listed in the NCBI non-redundant protein database (http://www.ncbi.nlm.nih.gov/protein/). Nevertheless, the new data on *C. elegans col-99* alternative splicing suggest that complex combinations of exon deletion are a common phenomenon in MACIT collagen genes.

To characterize the *C. elegans* COL-99 protein, we generated the fosmid-based *col-99::egfp::flag* worm line, CHO cells expressing COL-99, and for purposes of comparison, also cells expressing human collagen XIII, as well as two *C. elegans* COL-99-specific antibodies. Our experiments with the transfected CHO cells demonstrated that *col-99* encodes a protein of 85 kD, perfectly matching in size with human collagen XIII produced in the same cells. Like human collagen XIII, COL-99 has the biochemical and localization properties of a plasma membrane-associated protein. Moreover, our results identify that the predicted furin cleavage sites in the COL-99 NC1 domain are used by furin-like proteases for shedding of the ectodomain. We also identified in the worms a trimeric protein corresponding in size to that expected for the col-99::egfp::flag transgene.

The mRNA and protein levels of collagens XIII/XXIII/XXV in human and mouse tissues and cells are generally low [5, 6, 53–56]. However, increased expression of collagens XIII and XXIII are detected during cancer development [12, 13, 56]. Data from a systematic developmental gene expression time-course study suggest that mRNA levels of *col-99* in *C. elegans* are also low [57, 58]. The microarray data suggested slight up-regulation of the *col-99* mRNA in the embryonic developmental stages of ventral enclosure, embryo movement and L1 larva. This was also observed in the related species including *C. remanei*, *C. briggsae*, and *C. brenneri* [59].

We used the new *col-99::egfp::flag C. elegans* line to examine the tissue localization of COL-99 in the animals. Since the fosmid-based expression is under endogenous *cis* regulatory control by way of including introns and 5’and 3’ UTR sequences [24], the expression patterns of COL-99-EGFP-FLAG in *C. elegans* should correspond well with those of the endogenous protein. In larvae and adult animals, COL-99 expression was detected in mouth, pharynx, body wall and the tail, mostly in motor neurons and muscle. Especially notable was the co-localization of COL-99::EGFP::FLAG in adult worms and embryos with the NMJ marker α-bungarotoxin. It should be noted that in the late three-fold stage the worm can move inside the egg by rolling around its longitudinal axis, indicating advanced motor system development (http://www.wormatlas.org/).

Studies of MACIT collagens in humans and mice have suggested that these collagens have pathophysiological roles in the nervous system, in developmental innervation of muscles and at the NMJ [9, 11]. The expression of COL-99::EGFP::FLAG at the NMJ in worm embryos together with the neuromuscular phenotypes of collagen XIII and collagen XXV knockout mice suggests that MACIT collagens have conserved roles in NMJ or neuromuscular development.

## Conclusions

We identify that the MACIT transmembrane collagens are widespread in bilaterians, yet have been lost from multiple lineages. We infer that MACITs originated in the last bilaterian common ancestor, thus evolved far later than the fibrillar collagens. The collagen XIII/XXIII/XXV gene family members are encoded by paralogous genes that likely originated from the two rounds of *en-bloc* genome duplication early in vertebrate evolution. The data on *C. elegans* MACIT demonstrate conserved molecular properties and tissue localisations.

The combined novel insights, tools and dataset of protein sequences reported here, set up an important basis for further work. It will be important to identify the genetic interactors and functional protein ligands of COL-99, to accelerate our general understanding of MACIT collagens and especially of molecular mechanisms relevant to human diseases related to collagens XIII, XXIII and XXV.

## Methods

### Assembly of a dataset of collagens homologous to collagens XIII, XXIII and XXV

The C-terminal non-collagenous sequences (NC4) of human and mouse collagen XIII [4] were used initially to identify homologues in other species by BLASTP searches at NCBI. Homologous sequences were identified in *C. elegans* and *D. melanogaster*. Further homologues were identified by BLASTP and TBLASTN searches with the protein sequences of the relevant collagens from human, *C. elegans* and *D. melanogaster* (GenBank accessions: human collagen XIII, NP_001123575.1; human collagen XXIII, NP_775736.2; human collagen XXV, NP_942014.1; *D. melanogaster* collagen CG43342, NP_001138061 (UniProKB accession number B7Z0K8, gene ID 7354466, FlyBase ID FBgn0259244); *C. elegans* COL-99, NP_001122775.2) against the NCBI non-redundant protein and nucleotide databases at default parameters and, as needed, with use of Entrez terms to search individual phyla or classes of animals. Additional databases of invertebrate genomes and transcriptomes searched were Compagen (http://compagen.zoologie.uni-kiel.de/index.html) [60], Echinobase (http://mandolin.caltech.edu/Echinobase/) [61], and the Japanese Lamprey Genome Project (http://jlampreygenome.imcb.a-star.edu.sg/) [32]. Sequences returned with an E-value <1e-20 and sequence identity spanning the length of the protein were retained for further evaluation. Each identified protein sequence was validated as a MACIT by: A) confirming that the best reciprocal BLAST hits corresponded to human transmembrane collagens XIII, XXIII and XXV (E-value <1e-10, most E-values are <1e-40). B) Domain analysis by InterProScan (http://www.ebi.ac.uk/Tools/pfa/iprscan5/) and detailed inspection of sequence features, especially of the C-terminal region. Predicted protein orientation and transmembrane domains were identified through the resources of the Center for Biological Sequence Analysis, Technical University of Denmark (http://www.cbs.dtu.dk/services/). Coiled-coil regions were identified by MARCOIL (http://toolkit.tuebingen.mpg.de/marcoil). Predicted furin cleavage sites in MACITs were identified by PrOP 1.0 [37]. C) Additional TBLASTN searches against the NCBI databases of expressed sequence tags (dbest), or transcriptome shotgun assembly (TSA) were used to obtain evidence for transcription of identified open reading frames and were used in some cases to correct the GenBank predicted protein sequences. D) Construction of phylogenetic trees to validate the clade placement of newly identified MACIT sequences.

### Multiple sequence alignments and phylogenetic analysis

From the above identifications, a dataset of MACIT sequences was compiled that included representative sequences from all the phyla of animals in which MACITs had been identified (Table 1). Protein sequences were aligned in MUSCLE 3.8 [46] or webPRANK [45] through the online resources of EBI (www.ebi.ac.uk). Phylogenetic analysis based on these alignments was carried out in PhyML 3.0 [62], either without or with gap removal, using the LG amino acid substitution model [63] and 200 bootstrap cycles through the resources of phylogeny.france (http://phylogeny.lirmm.fr/phylo_cgi/index.cgi/). Tree-rendering of the Newick output was conducted in iTOL (Interactive Tree Of Life, itol.embl.de) [64].

### Analysis of paralogy of MACIT-encoding genes within the human genome

Paralogy between the human *COL13A1*, *COL23A1* and *COL25A1* genes was assessed initially with the database of “paralogons in the human genome” v5.28 in which searches are made based on chromosomes or chromosomal band regions (http://wolfe.ucd.ie/dup/human5.28/). Chromosomal locations of the three genes were then identified by TBLASTX searches of the human genome reference assembly scaffolds at NCBI (http://blast.ncbi.nlm.nih.gov/Blast.cgi, version GRCh38.p2). For each gene, local paralogous genes were identified on the NCBI human genome Map Viewer, annotation release 104, and its associated tools.

### Cloning and expression of human COL13-EGFP and *C. elegans col-99* cDNA in CHO cells

Cloning of human collagen XIII (COL13)-EGFP fusion cDNA into the mammalian cell expression vector pc3.1/Hygromycin (+) (Invitrogen) was carried out in 3 steps. 1. COL13 cDNA was produced using 1 μg of total RNA extracted from a mesenchymal prostate cell line EPT1 [65], and a M-MuLV reverse transcriptase kit (Thermo Scientific) followed by PCR amplification using the COL13_forward primer containing a HindIII cleavage site and the COL-13_reverse primer containing a XhoI site (Additional file 5). A 2 kb DNA fragment was extracted and purified from an agarose gel, and then digested with HindIII and XhoI (New England Biolabs), followed by insertion into the pcDNA3.1/Hygro (+) vector pre-cleaved with HindIII and XhoI. The construct was verified by HindIII/XhoI digestion and DNA sequencing. 2. The COL13 cDNA was cleaved with MfeI into two DNA fragments, 1.8 kb from the 5’ end and 0.2 kb from the 3’ end. The 3’ part together with an EGFP cDNA [66] were fused by two-step overlapping PCRs using primers as previously described for a COL13 cDNA from a human brain cDNA library fused with mCherry cDNA [67]. 3. The DNA fragments HindIII-COL13 (1.8 kb)-MfeI and MfeI-COL13 (0.2 kb)-EGFP-XhoI were ligated into the vector pcDNA3.1/Hygro (+) pre-cleaved by HindIII and XhoI. Due to the homologous sequences of mCherry and EGFP at both termini of the cDNA, all the primers for mCherry fusion cloning [67] also worked for EGFP.

To clone *col-99* cDNA, two μl of RT-reaction products from the worm line *col-99::egfp::flag* (for details of the worm line, see the following section of the Methods) RNA extract were used as a template in a PCR using a Q5 High-Fidelity DNA Polymerase kit (New England BioLabs) according to the manufacture’s instruction. The primers were col-99_5’ containing a HindIII cleavage site, and col-99_3’ containing an XhoI cleavage site (Additional file 5). A 2.3 kb PCR product was purified from an agarose gel and inserted into the HindIII and XhoI sites of the mammalian expression vector pc3.1 (+)/hygromycin using T4 ligase (Thermo Scientific). The ligation product was transformed to the *E. coli* strain DH5α, the insert sizes were verified by HindIII and XhoI cleavage, and the whole cDNA was confirmed by sequencing using the primers pc3.1_forward, col-99_middle, and pc3.1_reverse (Additional file 5).

To characterize the proteins huCOL13-EGFP and COL-99, the plasmid pc3.1(+)/Hygromycin/COL13-EGFP or pc3.1(+)/Hygromycin/col-99 was transfected into CHO cells (CHO-K1, ATCC® CCL-61^TM^, ATCC, Manassas, VA) using the TurboFect reagent (Thermo Scientific). The same vector with an inserted EGFP cDNA was used as a transfection efficiency control. Single cell clones with stable transfection were selected and amplified by hygromycin-resistance. To analyse COL-99 shedding, the cell culture medium was replaced with serum-free DMEM and the cell culture continued for another 2 days with daily addition of ascorbate (80 μg/ml). Proteins in the media were enriched by precipitation in 50 % methanol at -20 °C, pelleted by centrifugation and dissolved in SDS-PAGE sample buffer. The cells were treated with the same amount of SDS-PAGE sample buffer. To study the subcellular localization of COL-99 by western blotting, 5x10^6^ CHO/COL-99 cells were harvested in 1 ml of PBS containing Complete Protease Inhibitor Cocktail (Roche) and treated 5 times by freeze-thawing. After centrifugation at 16000 g for 5 mins, the supernatants, containing PBS-soluble proteins, were removed for further analyses. The pellets were extracted with 1 ml of 70 mM Tris, 300 mM NaCl, pH 7.4, containing 1 % Triton X-100 and the same protease inhibitors, by homogenization on ice. The Triton-soluble proteins were isolated by centrifugation under the same conditions. Twenty five μl of the sample from each fraction was applied for SDS-PAGE analysis. For western blotting, the human collagen XIII-EGFP fusion protein and the control protein EGFP were detected by GFP antibody. The COL-99 protein was detected by rabbit anti-COL-99 serum AB5625.11 (antigen peptide DQLPSSDSNTDDDD, custom made by Sigma-Genosys Ltd) and AB693 (antigen peptide LVAPNGTINEDLKK, custom made by Innovagen), each at 1:1000 dilution. To test the shedding of the COL-99 ectodomain by furin-like protease, Furin Inhibitor I (Calbiochem) in 10 μl methanol at final concentrations of 0, 1, 2, 5, or 10 μM was added daily to CHO/COL-99 cells at ~80 % confluency growing in a 12-well plate with 1 ml of serum-free culture medium per well. The media and cells were harvested 48 h post-treatment, the proteins in the medium were enriched as above, pelleted by centrifugation, and dissolved in 100 μl SDS-PAGE sample buffer. Cells were lysed directly in 100 μl SDS-PAGE sample buffer. Twenty five μl of each sample was loaded onto SDS-PAGE gels. β-tubulin was used as a loading control for the proteins from inhibitor-treated and non-treated cell lysates. To confirm the cell membrane localization of COL-99 protein, CHO/COL-99 cells were fixed with 4 % PFA for 30 min and, after a blocking step, stained with the antibody AB5625.11. AlexaFluor 594-conjugated donkey anti-rabbit IgG (Life Technologies) was used as a secondary antibody for the detection. Staining with the secondary antibody only was included as a negative control. Staining was imaged with a FluoView FV 1000 (Olympus) confocal microscope using a 100X objective.

### Generation of worm lines expressing EGFP- and FLAG-tagged COL-99 and PAT-3 proteins

The fosmid clones WRM0624B_B09 expressing the MACIT collagen *col-99::gfp::flag* and WRM0619C_E11 expressing integrin *pat-3::gfp::flag*, used as a control, were obtained from the *C. elegans* TransgeneOme project [24]. These constructs encode proteins with EGFP and FLAG tags at the C-termini. The fosmid clone selection and DNA production were performed according to the protocols provided by TransgeneOme [24], and the DNA sequences were validated using an ABI3500xL Genetic Analyzer (Life Technologies)

Ballistic transformation was prepared by microparticle bombardment into *C. elegans* strain HT1593 [*unc119 (ed3)III*, Caenorhabditis Genetics Center, University of Minnesota, Twin Cities]. The fosmids obtained from TransgeneOme contain an *unc-119* marker cassette which is used for transgene screening by rescuing loss-of-function mutations in the *C. elegans* strain HT1593 [*unc119 (ed3)III*]. The bombardment was performed according to the protocol described in [68]. Shortly, L1-L3 nematodes were picked and grown on enriched peptone plates seeded with *E. coli* C600 (CGSC, Coli Genetic Stock Center, Yale). The worms were bleached, and the eggs were isolated and grown at 20 °C to produce the worms suitable for bombardment. The microcarrier beads for the DNA transfer were prepared as follows: 30 mg of golden microparticles (0.3-3 μm particles, Chempur, Karlsruhe, Germany) were washed thoroughly with 70 % ethanol and sterile water sequentially and re-suspended in 500 μl of sterile 50 % glycerol to form a gold stock solution (60 mg/ml). The golden beads were coated by mixing gently for 30 min 10 μl of DNA (~5 μg) with 100 μl of gold stock solution, 100 μl of 2.5 M CaCl_2_ and 40 μl of 0.1 M Spermidine (Sigma-Aldrich), followed by washing in ethanol, and re-suspension in 170 μl of 100 % ethanol. Gene bombardment was carried out with a PDS-1000/He (Bio-Rad) using 1350 psi rupture disks according to the manufacture’s protocol. After the transformation, targeted nematodes with a phenotype rescued by the marker cassette were picked after 2 weeks based on their normal movement, and the lines were further cultured. Nematodes were maintained on NGM plates at 20 °C with the *E. coli* strain OP50 as a food source [69].

### Verification of the transgenic worm lines *col-99::egfp::flag* and *pat-3::egfp::flag*

To verify the worm lines *col-99::egfp::flag* and *pat-3::egfp::flag*, ten adult *C. elegans* worms from each gene bombardment line with normal movement were transferred to 100-mm diameter NGM/OP50 plates and cultured at 20 °C for 4 days. Animals (of mixed stages) were washed thoroughly with M9 buffer containing 22 mM KH_2_PO_4_, 42 mM Na_2_HPO_4_, 90 mM NaCl, and 1 mM MgSO_4_, pH 7.0, and then disrupted in 1 ml of TriReagent (Sigma-Aldrich) by 7 repeated freeze-thaw treatments. RNA was separated from DNA and protein by phase separation with chloroform, and then purified by a Qiagen RNeasy Mini Kit (Qiagen). RNA quality was measured by RNA Integrity Score (RIS) using an automatic QIAxcel RNA QC kit v2.0 (Qiagen). One μg of RNA with RIS ≥ 7 was used for cDNA synthesis in 50 μl using the First Strand cDNA Synthesis kit (Thermo Scientific) according to manufacturer’s protocol. One μl from the Reverse Transcriptase (RT) -reaction was used as a template for PCR using Taq DNA polymerase (Thermo Scientific) and the primers GFPinternal_forward and GFPinternal_reverse (Additional file 5) that were also used for DNA sequence validation. The PCR products were analyzed on agarose gels and visualized by Midori Green DNA Stain (Nippon Genetics) on a Gel Doc™ XR^+^ System (Bio-Rad). The primers for the reference gene *tba-1* are tba-1_forward and tba-1_reverse, and for *col-99,* col-99_forward and col-99_reverse (Additional file 5).

The expression of the recombinant fusion protein in the targeted worm lines was analyzed by western blotting. Total worm protein was prepared using the TriReagent according to the manufacture’s instruction. The protein pellets were dissolved in 1 % SDS and the total protein amount was measured with Direct Detect (Millipore). Seventy μg of total protein per lane was applied to SDS-PAGE. In some experiments, the primary antibody was mouse anti-FLAG (Sigma Aldrich) and the secondary antibody was HRP-conjugated goat anti-mouse IgG (Sigma Aldrich), and in other experiments the primary antibody was rabbit anti-GFP (Rockland Immunochemicals), with HRP-conjugated goat anti-rabbit as the secondary antibody (Jackson ImmunoResearch Inc., PA, USA). The total protein loading per lane was calibrated on β-tubulin, detected by a monoclonal antibody recognizing β-tubulin in all eukaryotic cells (Thermo Scientific). An ECL Prime Western Blotting Detection Reagent (GE Lifesciences) was used for signal detection and the imaging was processed with ImageQuant LAS 3000 (GE Lifesciences).

### Worm imaging

Animals at mixed stages were anesthetized with 10 mM sodium azide in M9 buffer for 1 h, mounted onto 2 % agarose pads freshly prepared on microscopy glass slides, and examined immediately using LSM 780 (Zeiss) or FluoView FV 1000 (Olympus) confocal microscopes.

For whole-mount immunofluorescence staining, the worms were transferred from NGM/OP50 culture plates and washed with M9 solution. After freezing the samples at -80 °C overnight, the animals were thawed on ice and then fixed with 0.5 ml of cold methanol at 4 °C for 10 mins, and partly disrupted by sonication twice with Digital Sonifier Models 450 (Branson Ultrasonics Corporation) at 65 % amplitude for 5 s. After being settled on ice for 10 mins, the pellets were spun down and treated with 0.5 ml cold acetone on ice for 10 min, followed by washing firstly with 0.5 ml of PBS containing 0.5 % BSA and 0.05 % Tween-20, and then with the same solution containing 20 % glycerol for another 30 min. The specimens were then blocked with PBS containing 2 % BSA, 0.2 % gelatin, 2 % fat-free milk and 0.05 % Tween-20, at room temperature for 2 h. The primary and secondary antibodies were diluted in blocking solution and incubated with the worms at room temperature for 2 h or at 4 °C overnight. For double staining, the primary antibodies were incubated one by one. The antibodies were used at the following dilutions: rabbit anti-GFP (Molecular Probes) 1:1000, mouse anti- *C. elegans* myosin heavy chain A (DSHB, Developmental Studies Hybridoma Bank) 1:20, AlexaFluor conjugated donkey anti-rabbit IgG (H + L), donkey anti-mouse IgG (H + L), and bungarotoxin (Molecular Probes) 1:1000. After staining, the worms were transferred to glass slides with 10 μl of DuoLink In Situ Mounting Medium with DAPI (Sigma-Aldrich), covered with glass cover slips and sealed with nail polish. The staining was analyzed on a FluoView FV 1000 confocal microscope (Olympus) or LSM 780 confocal microscope (Zeiss) using a 100X objective. Image reconstruction and merges were obtained in Zen Lite (Zeiss) or Image J (NIH). Specimens were also prepared on poly-L-lysine coated glass slides using a freeze-crack protocol [70], and the samples were fixed with 4 % PFA at 4 °C for 2 h. The conversion of red fluorescence to magenta and new color merging were performed in Corel PaintShop Photo ProX3 (Corel).

### Ethics statement

No vertebrate animals were used for these studies and no ethical approval was required.

### Availability of supporting data

The COL-99 isoform f (col-99) mRNA sequence is available in GenBank with the accession number of KM875546. Table 1 lists the protein sequences on which the phylogenetic study is based and states the GenBank accession number for each protein sequence.

## Additional files

Additional file 1:
**Protein sequence alignment of human collagens XIII, XXIII, XXV and six alternative spliced variants of COL-99.** The protein sequence of the newly identified COL-99f was compared with the other COL-99 variants and human collagens XIII, XXIII and XXV. Putative furin cleavage residues in these proteins and the peptides for producing the COL-99 antibodies AB5625.11 and AB693 are highlighted in the sequence. (PDF 22 kb) Additional file 2:
**Exon-intron alignment of COL-99 variants.** All the six COL-99 variants are subject to alternative splicing affecting different exons. The newly identified COL-99f lacks exons 4, 12, 16. (PDF 80 kb) Additional file 3:
**Western blot analysis of COL-99::EGFP::FLAG expression in** ***C. elegans*** **with anti-GFP or anti-FLAG antibodies.** This supplemental figure indicates that both anti-GFP and anti-FLAG are able to detect COL-99::EGFP::FLAG protein in the *C. elegans* worm lysates, but compared to the anti-FLAG, the anti-GFP antibody detects non-specific bands. (PDF 141 kb) Additional file 4:
**Expression of PAT-3::EGFP::FLAG in** ***C. elegans*** **.** This supplemental figure shows strong in vivo GFP signals in worm muscles and other tissues verifying the strategy and technique in the fosmid-based transgenic worm generation. (PDF 141 kb) Additional file 5:
**A table listing the PCR primers used in this study.** All the PCR primer sequences for col-99f cDNA and human collagen XIII cDNA with EGFP tag cloning, transgenic worm line verification, and RT-PCR are provided in the table. (PDF 39 kb)

## Abbreviations

ECM: extracellular matrix
MACIT: membrane-associated collagens with interrupted triple-helices
NMJ: neuromuscular junction
