# Supplementary material for: Membrane-associated collagens with interrupted triple-helices (MACITs): evolution from a bilaterian common ancestor and functional conservation in C. elegans
Source: BMC Evol Biol. 2015 Dec 14;15:281. doi: 10.1186/s12862-015-0554-3 (PMC4678570; doi:10.1186/s12862-015-0554-3)
Supplement: Additional file 1: — Protein sequence alignment of human collagens XIII, XXIII, XXV and six alternative spliced variants of COL-99. The protein sequence of the newly identified COL-99f was compared with the other COL-99 variants and human collagens XIII, XXIII and XXV. Putative furin cleavage residues in these proteins and the peptides for producing the COL-99 antibodies AB5625.11 and AB693 are highlighted in the sequence. (PDF 22 kb) [file 12862_2015_554_MOESM1_ESM.pdf]

Additional files

Additional file 1. Protein sequence alignment of human collagens XIII, XXIII, XXV and six alternative spliced variants of COL-99. The protein sequence identified and partially characterized in this study is COL-99f. Multiple sequence alignment was performed using Clustal Omega. The output of the alignment was based on the input order but not the aligned one. Asterisks indicate the same residues in all the sequences. Two dots indicate high similarity of amino acid chemical properties, and one dot indicates less similarity. Cysteines are highlighted in yellow, alternative splicing sites are highlighted in green, putative furin cleavage sites are in bold red, and the peptides for COL-99 antibody production are underlined.

```
HsXIII       MVAERTHK-------AAATGARGPGELG--AP---GTVALVAARAERGARLPSPGSCGLL    48
HsXXIII      MGPGERAGGGGDAGKGNAAGGGGGGRS----A---T-------------TAGSRAVSAL    39
HsXXV        MLLKKH-----------A-GKGGGRE----P---RSEDPT--PAEQHC-ARTMPPCAVL    37
COL-99a      MTSPSP------SGNVVVVGTDGTSSVSDRWPPQKTWISPPRVPIDRHFVTAA-------    47
COL-99b      MTSPSP------SGNVVVVGTDGTSSVSDRWPPQKTWISPPRVPIDRHFVTAA-------    47
COL-99c      MTSPSP------SGNVVVVGTDGTSSVSDRWPPQKTWISPPRVPIDRHFVTAA-------    47
COL-99d      MTSPSP------SGNVVVVGTDGTSSVSDRWPPQKTWISPPRVPIDRHFVTAA-------    47
COL-99e      MTSPSP------SGNVVVVGTDGTSSVSDRWPPQKTWISPPRVPIDRHFVTAA-------    47
COL-99f      MTSPSP------SGNVVVVGTDGTSSVSDRWPPQKTWISPPRVPIDRHFVTAA-------    47
             *                 .   * .

HsXIII       TLA--LCSLALSLLAHFRTAE-LARVLRLEAERGEQ---------QMETAILGRVNQLL    95
HsXXIII      CLLLSVGSAAACLLLGVQAAALQGRVAALEEERELLRRAGPPGALD---AWAEPHLERLL    96
HsXXV        AALLSVVAVVSCLYLGVKTNDLQARIAALESAKGAPSIHLLPDTLDHLKTMVQEKVERLL    97
COL-99a      -VPHVLMFLLVCIVF----TAQQTRISTLEKRI------------DQL----VVQIDQLP    86
COL-99b      -VPHVLMFLLVCIVF----TAQQTRISTLEKRI------------DQL----VVQIDQLP    86
COL-99c      -VPHVLMFLLVCIVF----TAQQTRISTLEKRI------------DQL----VVQIDQLP    86
COL-99d      -VPHVLMFLLVCIVF----TAQQTRISTLEKRI------------DQL----VVQIDQLP    86
COL-99e      -VPHVLMFLLVCIVF----TAQQTRISTLEKRI------------DQL----VVQIDQLP    86
COL-99f      -VPHVLMFLLVCIVF----TAQQTRISTLEKRI------------DQL----VVQIDQLP    86
                  :     .:           *:  **                        ::::*

HsXIII       DEKWKLHSRRRREAPKTSPGCNCPPAFQGPTGRPGLPGDKGAIGMPGRVGSPG-------    148
HsXXIII      -REKLDGLAKIRTAREAPSECVCPPGPPGRRGKPGRRGD--------------------    134
HsXXV        AQKSYEHMAKIRIAREAPSECNCPAGPPGKRGKRGRRGE--------------------    136
COL-99a      SSDSNTDDDDVAKSRRVRNSCMCPAGPPGERGPVGPPG---------LPGLPAPYYRRPR    137
COL-99b      SSDSNTDDDDVAKSRRVRNSCMCPAGPPGERGPVGPPGLRGSPGWPGLPGLPAPYYRRPR    146
COL-99c      SSDSNTDDDDVAKSRRVRNSCMCPAGPPGERGPVGPPG---------LPGLPAPYYRRPR    137
COL-99d      SSDSNTDDDDVAKSRRVRNSCMCPAGPPGERGPVGPPG---------LPGLPAPYYRRPR    137
COL-99e      SSDSNTDDDDVAKSRRVRNSCMCPAGPPGERGPVGPPG---------LPGLPAPYYRRPR    137
COL-99f      SSDSNTDDDDVAKSRRVRNSCMCPAGPPGERGPVGPPG---------LPGLPAPYYRRPR    137
               .          : ..   * ** .  *  *  *  *

HsXIII       -------------------------DAGL---SIIGPRGPPGQPGTRGFPGFPGPIGLDG    180
HsXXIII      ----------------------------------------PGPPGQSGRDGYPGPLGLDG    154
HsXXV        ----------------------------------------SGPPGQPGPQGPPGPKGDKG    156
COL-99a      VPLSNNLDESISRKMRAFGMLYSPDGQAIQLRGMPGPPGPAGPKGLRGYPGFPGPIGLDG    197
COL-99b      VPLSNNLDESISRKMRAFGMLYSPDGQAIQLRGMPGPPGPAGPKGLRGYPGFPGPIGLDG    206
COL-99c      VPLSNNLDESISRKMRAFGMLYSPDGQAIQLRGMPGPPGPAGPKGLRGYPGFP-------    190
COL-99d      VPLSNNLDESISRKMRAFGMLYSPDGQAIQLRGMPGPPGPAGPKGLRGYPGFPGPIGLDG    197
COL-99e      VPLSNNLDESISRKMRAFGMLYSPDGQAIQLRGMPGPPGPAGPKGLRGYPGFPGPIGLDG    197
COL-99f      VPLSNNLDESISRKMRAFGMLYSPDGQAIQLRGMPGPPGPAGPKGLRGYPGFPGPIGLDG    197
                                                      *  *  *  * *

HsXIII       KPGHPGPKG------DMGLTGPPGQPGPQGQKGEKGQCGEYPHRECLSSMPAALRSSQII    234
HsXXIII      KPGLPGPKG------EKGA-----------------------------------------    167
HsXXV        EQGDQGPRM------VFPKI----------------------------------------    170
COL-99a      PRGLPGTPGSKGDRGERGPLGPPGFPGPKGDRGVMTG--PYVGPH---------------    240
COL-99b      PRGLPGTPGSKGDRGERGPLGPPGFPGPKGDRGVMTG--PYVGPH---------------    249
```

```
COL-99c      -----GTPGSKGDRGERGPLGPPGFPGPKGDRGVMTG--PYVGPH---------------   228
COL-99d      PRGLPGTPGSKGDRGERGPLGPPGFPGPKGDRGVMTG--PYVGPH---------------   240
COL-99e      PRGLPGTPGSKGDRGERGPLGPPGFPGPKGDRGVMTG--PYVGPH---------------   240
COL-99f      PRGLPGTPGSKGDRGERGPLGPPGFPGPKGDRGVMTG--PYVGPH---------------   240
                  *

HsXIII       ALKLLPLLNSVRLAPPPVIKRRTFQGEQSQASIQGPPGPPGPPGPSGPLGHPGLPGPMGP   294
HsXXIII      ---------------PGDFGPRGDQGQDGAAG---PPGPPGPPGARGPPGDTGKDGPRGA   209
HsXXV        -------NHGFLSADQQLIKRRLIKGDQGQAG---PPGPPGPPGPRGPPGDTGKDGPRGM   220
COL-99a      -------------AGPGPMSHH----TNMGNVLPGPPGPPGPPGPAGRDGRHGLKGDRGL   283
COL-99b      -------------AGPGPMSHH----TNMGNVLPGPPGPPGPPGPAGRDGRHGLKGDRGL   292
COL-99c      -------------AGPGPMSHH----TNMGNVLPGPPGPPGPPGPAGRDGRHGLKGDRGL   271
COL-99d      -------------AGPGPMSHH----TNMGNVLPGPPGPPGPPGPAGRDGRHGLKGDRGL   283
COL-99e      -------------AGPGPMSHH----TNMGNVLPGPPGPPGPPGPAGRDGRHGLKGDRGL   283
COL-99f      -------------AGPGPMSHH----TNMGNVLPGPPGPPGPPGPAGRDGRHGLKGDRGL   283
                               :  :     :       *********  *  *  *  *  *

HsXIII       PGLPG-------------------------PPGPKGDPGIQGYHGRKGERGMPGMP---   325
HsXXIII      QGPAGPK--------GEPGQDGEMGPKGPPGPKGEPGVPG-------------------   241
HsXXV        PGVPGEP--------GKPGEQGLMGPLGPPGQKGSIGAPGIPGMNGQKGEPGLPGAVGQN   272
COL-99a      PGFDGESKIGPKGETGSPGRDGIPGARGPPGERGEKGDTAFLSTYPRVASSST------A   337
COL-99b      PGFDGESKIGPKGETGSPGRDGIPGARGPPGERGEKGDTAFLSTYPRVASSST------A   346
COL-99c      PGFDGESKIGPKGETGSPGRDGIPGARGPPGERGEKGDTAFLSTYPRVASSST------A   325
COL-99d      PGFDGESKIGPKGETGSPGRDGIPGARGPPGERGEKGDTAFLSTYPRVASSST------A   337
COL-99e      PGFDGESKIGPKGETGSPGRDGIPGARGPPGERGEKGDTAFLSTYPRVASSST------A   337
COL-99f      PGFDGESKIGPKGETGSPGRDGIPGARGPPGERGEKGDTAFLSTYPRVASSST------A   337
              *  *                            *  *  .

HsXIII       ------------------------GKHGAKGAPGIAVAGMKGEPGIPGTKGEKGAEGSPG   361
HsXXIII      ---------------------------------------------------KKGDDGTPS   250
HsXXV        GIPGPKGEPGEQGEKGDAGENGPKGDTGEKGDPGSSAAGIKGEPGESGRPGQKGEPGLPG   332
COL-99a      SSPGPPGPPGPP---GVCHASQCTGIQGPPGEPGRTIIGPQGPPGEKGERGERGEPGDRG   394
COL-99b      SSPGPPGPPGPP---GVCHASQCTGIQGPPGEPGRTIIGPQGPPGEKGERGERGEPGDRG   403
COL-99c      SSPGPPGPPGPP---GVCHASQCTGIQGPPGEPGRTIIGPQGPPGEKGERGERGEPGDRG   382
COL-99d      SSPGPPGPPGPP---GVCHASQCTGIQGPPGEPGRTIIGPQGPPGEKGERGERGEPGDRG   394
COL-99e      SSPGPPGPPGPP---GVCHASQCTGIQGPPGEPGRTIIGPQGPPGEKGERGERGEPGDRG   394
COL-99f      SSPGPPGPPGPP---GVCHASQCTGIQGPPGEPGRTIIGPQGPPGEKGERGERGEPGDRG   394
                                                                ::*  *  .

HsXIII       LPGLLGQKGEKGDAGNSIGGGRGEPGPPGL--------------------PGPPGPKGEA   401
HsXXIII      QPGPPGPKGEPGSMG-----PRGENGVDGAPGP--------------------------   278
HsXXV        LPGLPGIKGEPGFIG-----PQGEPGLPGLPGTKGERGEAGPPGRGERGEPGAPGPKGKQ   387
COL-99a      LPGAAG---AAN----LLNGGK-------------------------ALVGPPGPP---   418
COL-99b      LPGAAG---AAN----LLNGGK-------------------------ALVGPPGPP---   427
COL-99c      LPGAAG---AAN----LLNGGK-------------------------ALVGPPGPP---   406
COL-99d      LPGAAG---AAN----LLNGGK-------------------------ALVGPPGPP---   418
COL-99e      LPGAAG---AAN----LLNGGK-------------------------ALVGPPGPP---   418
COL-99f      LPGAAG---AAN----LLNGGK-------------------------ALVGPPGPP---   418
              **  *               :

HsXIII       GVDGQVGPPGQPGDKGERGAAGEQGPDGPKGSKGEPGKG--EMVDYNGNINEALQEIRTL   459
HsXXIII      -----------KGEPGHRGTDGAAGPRGAPGLKGEQGDT--VVIDYDGRILDALKG----   321
HsXXV        GESGTRGPKGSKGDRGEKGDSGAQGPRGPPGQKGDQGAT--EIIDYNGNLHEALQRITTL   445
COL-99a      GRDGRPGDKGEKGEQGLRGDMGLPGPEGTPGKRGRRGRHGISLVAPNGTINEDLKKLLKT   478
COL-99b      GRDGRPGDKGEKGEQGLRGDMGLPGPEGTPGKRGRRGRHGISLVAPNGTINEDLKKLLKT   487
COL-99c      GRDGRPGDKGEKGEQGLRGDMGLPGPEGTPGKRGRRGRHGISLVAPNGTINEDLKKLLKT   466
COL-99d      GRDGRPGDKGEK------------------------GRHGISLVAPNGTINEDLKKLLKT   454
COL-99e      GRDGRPGDKGEKGEQGLRGDMGLPGPEGTPGKRGRRGRHGISLVAPNGTINEDLKKLLKT   478
COL-99f      GRDGRPGDKGEK------------------------GRHGISLVAPNGTINEDLKKLLKT   454
                                                 *     ::  :* : : *:

HsXIII       ALMGPPGLPGQIGPPGAPGIPGQKGEIGLPGPPGHDGEKGPRGKPGDMGPPGPQGPPGKD   519
HsXXIII      -PPGPQ------GPPGPPGIPGAKGELGLPGAPGIDGEKGPKGQKGDPGEPGPAGLKGEA   374
```

```
HsXXV        TVTGPP------GPPGPQGLQGPKGEQGSPGIPGMDGEQGLKGSKGDMGDPG---MTGEK    496
COL-99a      ELMPLL----------IEDISELRGKNVIPG------------------PPGPPGPRGHH    510
COL-99b      ELMPLL----------IEDISELRGKNVIPG------------------PPGPPGPRGHH    519
COL-99c      ELMPLL----------IEDISELRGKNVIPG------------------PPGPPGPRGHH    498
COL-99d      ELMPLL----------IEDISELRGKNVIPG------------------PPGPPGPRGHH    486
COL-99e      ELMPLL----------IEDISELRGKNVIPG------------------PPGPPGPRGHH    510
COL-99f      ELMPLL----------IEDISELRGKNVIPG------------------PPGPPGPRGHH    486
                                :   :*:   **                   **     *.

HsXIII       GPPGVKGENGHPGSPGEKGEKGETGQAGSPGEKGEAGEKGNPGAEVPGLPGPEGPPGPPG    579
HsXXIII      GEMG------LSGLPGADGLKGEKGESASDSLQE----SLAQLIVEPGPPGPPGPPGPMG    424
HsXXV        GGIG------LPGLPGANGMKGEKGDSGMPGPQ------GPSIIGPPGPPGPHGPPGPMG    544
COL-99a      GPVGPSGERGPQGLPGHSGERGDRGDIGPPGLPGQPGAGEISG-SQSG------PRGPP-    562
COL-99b      GPVGPSGERGPQGLPGHSGERGDRGDIGPPGLPGQPGAGEISG-SQSG------PRGPP-    571
COL-99c      GPVGPSGERGPQGLPGHSGERGDRGDIGPPGLPGQPGAGEISG-SQSG------PRGPP-    550
COL-99d      GPVGPSGERGPQGLPGHSGERGDRGDIGPPGLPGQPGAGEISG-SQSG------PRGPP-    538
COL-99e      GPVGPSGERGPQGLPGHSGERGDRGDIGPPGLPGQPGAGEISG-SQSG------PRGPP-    562
COL-99f      GPVGPSGERGPQGLPGHSGERGDRGDIGPPGLPGQPGAGEISG-SQSG------PRGPP-    538
             *  *        * ** .* :*: *: .  .                *      * **

HsXIII       LQGVPGPKGEAGLDGAKGEKGFQGEKGDRGPLGLPGASGLDGRPGPPGTPGPIGVPGPAG    639
HsXXIII      LQGIQGPKGLDGAKGEKGASGERGPSGLPGPVG---------------PPGLIGLPGTKG    469
HsXXV        PHGLPGPKGTDGPMGPHGPAGPKGERGEKGAMG---------------EPGP---RGPYG    586
COL-99a      --GLPGP---------------PGEKGDLGPPGLPGQP---GSLGLPGPPGPMGLRGPHG    602
COL-99b      --GLPGP---------------PGEKGDLGPPGLPGQP---GSLGLPGPPGPMGLRGPHG    611
COL-99c      --GLPGP---------------PGEKGDLGPPGLPGQP---GSLGLPGPPGPMGLRGPHG    590
COL-99d      --GLPGP---------------PGEKGDLGPPGLPGQP---GSLGLPGPPGPMGLRGPHG    578
COL-99e      --GLPGP---------------PGEKGDLGPPGLPGQP---GSLGLPGPPGPMGLRGPHG    602
COL-99f      --GLPGP---------------PGEKGDLGPPGLPGQP---GSLGLPGPPGPMGLRGPHG    578
               *: **                *  *  *  *                **     *  *

HsXIII       PKGERGSKGDPGMTGPTGAAGLPGLHGPPGDKGN------RGERGKKGSRGPKGDKGDQG    693
HsXXIII      EKGR---PGE---------PGLDGFPGPRGEKGDRSERGEKGERGVPGRKGVKGQKGEPG    517
HsXXV        LPGK---DGE---------PGLDGFPGPRGEKGDLGEKGEKGFRGVK---GEKGEPGQPG    631
COL-99a      TEGE------TGKQGP---EGSKGYPGPMGPQGPP---GNDGEPGIDGRPGPAGEKGDQG    650
COL-99b      TEGE------TGKQGP---EGSKGYPGPMGPQGPP---GNDGEPGIDGRPGPAGEKGDQG    659
COL-99c      TEGE------TGKQGP---EGSKGYPGPMGPQGPP---GNDGEPGIDGRPGPAGEKGDQG    638
COL-99d      TEGE------TGKQGP---EGSKGYPGPMGPQGPP---GNDGEPGIDGRPGPAGEKGDQG    626
COL-99e      TEGE------TG------------------PQGPP---GNDGEPGIDGRPGPAGEKGDQG    635
COL-99f      TEGE------TG------------------PQGPP---GNDGEPGIDGRPGPAGEKGDQG    611
               *.                           :*        *  *     *  *: *: *

HsXIII       APGLDAPCPLGEDGLPVQGCWNK----------------------------------       716
HsXXIII      PPGLDQPCPVGPDGLPVPGCWHK----------------------------------       540
HsXXV        LDGLDAPCQLGPDGLPMPGCWQK----------------------------------       654
COL-99a      IPGLDAPCPTGPDGLPLPYCSWKPMDGKNDVWERRKRASLPGAQPGKGAETRPPVTD       707
COL-99b      IPGLDAPCPTGPDGLPLPYCSWKPMDGKNDVWERRKRASLPGAQPGKGAETRPPVTD       716
COL-99c      IPGLDAPCPTGPDGLPLPYCSWKPMDGKNDVWERRKRASLPGAQPGKGAETRPPVTD       695
COL-99d      IPGLDAPCPTGPDGLPLPYCSWKPMDGKNDVWERRKRASLPGAQPGKGAETRPPVTD       683
COL-99e      IPGLDAPCPTGPDGLPLPYCSWKPMDGKNDVWERRKRASLPGAQPGKGAETRPPVTD       692
COL-99f      IPGLDAPCPTGPDGLPLPYCSWKPMDGKNDVWERRKRASLPGAQPGKGAETRPPVTD       668
               *** **  * ****:  *  *
```
