# Supplementary material for: Membrane-associated collagens with interrupted triple-helices (MACITs): evolution from a bilaterian common ancestor and functional conservation in C. elegans
Source: BMC Evol Biol. 2015 Dec 14;15:281. doi: 10.1186/s12862-015-0554-3 (PMC4678570; doi:10.1186/s12862-015-0554-3)

Additional file 2. Exon-intron alignment of COL-99 variants. Boxes in magenta indicate the exisiting exons and boxes in blue indicate the alternatively-spliced exons. Exons are numbered and lines indicate introns. The diagram is drawn to scale for the nucleotide lengths.

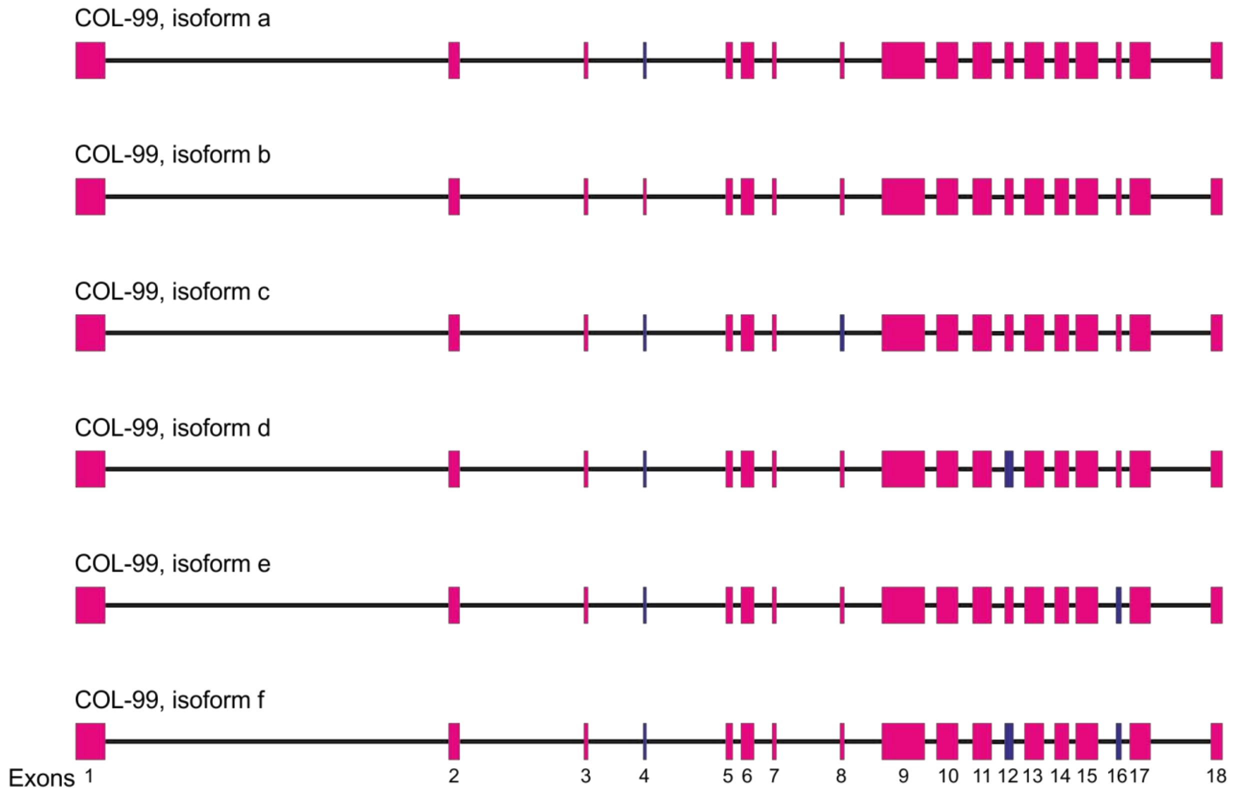

Supplement: Additional file 2: — Exon-intron alignment of COL-99 variants. All the six COL-99 variants are subject to alternative splicing affecting different exons. The newly identified COL-99f lacks exons 4, 12, 16. (PDF 80 kb) [file 12862_2015_554_MOESM2_ESM.pdf]
