# Supplementary material for: Membrane-associated collagens with interrupted triple-helices (MACITs): evolution from a bilaterian common ancestor and functional conservation in C. elegans
Source: BMC Evol Biol. 2015 Dec 14;15:281. doi: 10.1186/s12862-015-0554-3 (PMC4678570; doi:10.1186/s12862-015-0554-3)

Additional file 3. Western blot analysis of COL-99::EGFP::FLAG expression in *C. elegans* with anti-GFP or anti-FLAG antibodies. Samples were resolved under reducing conditions. The arrow indicates the band representing COL-99::EGFP::FLAG in the anti-GFP blot.

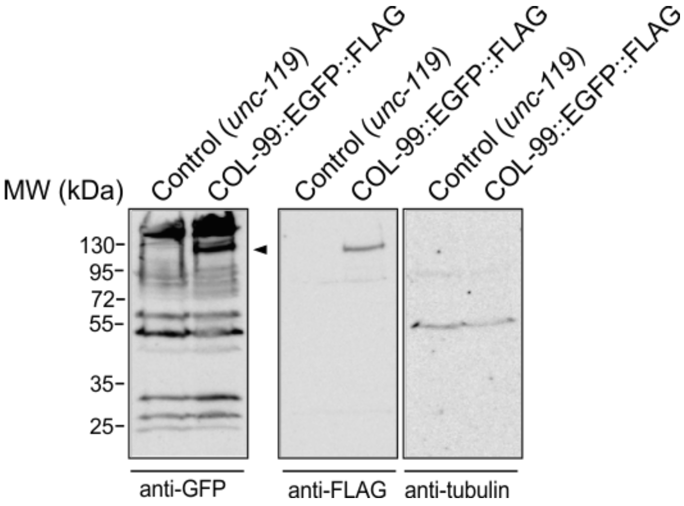

Supplement: Additional file 3: — Western blot analysis of COL-99::EGFP::FLAG expression in C. elegans with anti-GFP or anti-FLAG antibodies. This supplemental figure indicates that both anti-GFP and anti-FLAG are able to detect COL-99::EGFP::FLAG protein in the C. elegans worm lysates, but compared to the anti-FLAG, the anti-GFP antibody detects non-specific bands. (PDF 141 kb) [file 12862_2015_554_MOESM3_ESM.pdf]
