# Supplementary material for: Membrane-associated collagens with interrupted triple-helices (MACITs): evolution from a bilaterian common ancestor and functional conservation in C. elegans
Source: BMC Evol Biol. 2015 Dec 14;15:281. doi: 10.1186/s12862-015-0554-3 (PMC4678570; doi:10.1186/s12862-015-0554-3)

Additional file 4. Expression of PAT-3::EGFP::FLAG in *C. elegans*. (A) *In vivo* confocal imaging of *C. elegans* line *pat-3::egfp::flag*. (B) Detection of PAT-3::EGFP::FLAG in *C. elegans* muscle after a freeze-crack treatment and mild PFA fixation. The green fluorescence is generated by the EGFP-tag. Bar, 20 µm.

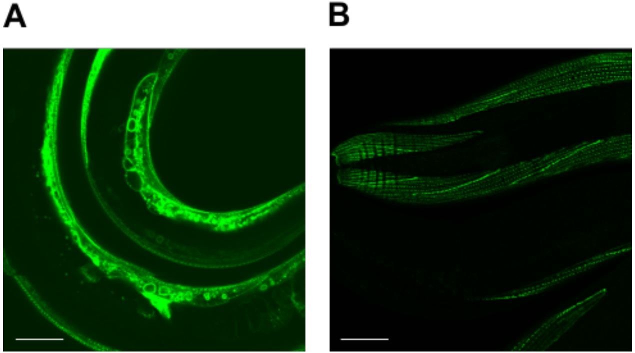

Supplement: Additional file 4: — Expression of PAT-3::EGFP::FLAG in C. elegans . This supplemental figure shows strong in vivo GFP signals in worm muscles and other tissues verifying the strategy and technique in the fosmid-based transgenic worm generation. (PDF 141 kb) [file 12862_2015_554_MOESM4_ESM.pdf]
