# Supplementary material for: Membrane-associated collagens with interrupted triple-helices (MACITs): evolution from a bilaterian common ancestor and functional conservation in C. elegans
Source: BMC Evol Biol. 2015 Dec 14;15:281. doi: 10.1186/s12862-015-0554-3 (PMC4678570; doi:10.1186/s12862-015-0554-3)
Supplement: Additional file 5: — A table listing the PCR primers used in this study. All the PCR primer sequences for col-99f cDNA and human collagen XIII cDNA with EGFP tag cloning, transgenic worm line verification, and RT-PCR are provided in the table. (PDF 39 kb) [file 12862_2015_554_MOESM5_ESM.pdf]

Additional File 5. List of PCR primers used in this study.

Primer name	DNA Oligonucleotide sequence, 5’ to 3’
GFP_reverse	CCATCTAATTCAACAAGAATTGGG
GFPinternal_forward	ACGGGAACTACAAGACAC
GFPinternal_reverse	GTTTGTCCGCCATGATGT
GFP_forward	GAGTTTGTAACAGCTGCTGGG
tba-1_forward	TCAACACTGCCATCGCCGCC
tba-1_reverse	TCCAAGCGAGACCAGGCTTCAG
col-99_forward	CCCCATCACCATCT
col-99_reverse	GAATCTGATGAAGGGAGTTGGTCTATCTG
COL13_forward	GGATAGAAGCTTTTGGCAGCGGCTGTCGCCT
COL-13_reverse	AATACTCGAGGTACAAACACACACACAGGCCAATC
col-99_5’	GGATAGAAGCTTGCCATGACCTCCCCATCACCA
col-99_3’	TCTAGACTCGAGTCAGTCAGTGACTGGCGGCCT
pc3.1_forward	TAATACGACTCACTATAGGG
col-99_middle	TAGAAGGCACAGTCGAGGCT
pc3.1_reverse	CAGCTGGAAGAGACGGAAGACAT
